# Integrated metagenomic and soil chemical analyses revealed shifts of microbial nutrient cycling with poplar plantation age

**DOI:** 10.3389/fpls.2025.1513281

**Published:** 2025-10-20

**Authors:** Yimin You, Xiaoting Liu, Liran Wang, Muhammad Khalid, Xuelai Wang, Luping Jiang, Fusen Wang, Zhongyi Pang, Yanhui Peng, Xiyang Zhao

**Affiliations:** ^1^ Jilin Provincial Key Laboratory of Tree and Grass Genetics and Breeding, College of Forestry and Grassland Science, Jilin Agricultural University, Changchun, China; ^2^ National Key Laboratory of Forest Genetics and Breeding, Northeast Forestry University, Harbin, China; ^3^ Department of Biology, College of Science, Mathematics and Technology, Wenzhou-Kean University, Wenzhou, China; ^4^ Qiqihar Branch, Heilongjiang Academy of Forestry Sciences, Heilongjiang, Qiqihar, China; ^5^ Forest Farm, State-owned Xinmin Mechanical Forest Farm, Xinmin, China

**Keywords:** plant-microbial interactions, microbiome, co-occurrence network, rhizosphere, nutrient cycling

## Abstract

**Introduction:**

Poplar (Populus spp.) is widely recognized as an ideal model system for studying plant-microbial interactions due to its rapid growth, genetic tractability, and ecological importance in afforestation programs. Leveraging these advantages, we investigated how poplar cultivation reshapes soil microbial communities and their nutrient cycling functions. Although plant roots are known to profoundly influence microbial community structure and functionality, comprehensive studies systematically linking poplar-induced microbiome shifts to nutrient cycling remain limited.

**Methods:**

Here, we employed an integrative approach combining metagenomic sequencing with soil nutrient analyses to assess poplar-induced changes in microbial community and metabolic activities at the root-soil interface.

**Results:**

Our analyses revealed three major findings: (1) poplar cultivation significantly altered the composition of microbial communities—including bacteria, fungi, and archaea—and reduced the complexity of microbial interaction networks, as revealed by co-occurrence analysis; (2) poplar cultivation enhanced microbial genetic potential related to degradation pathways for starch, lignin, and aromatic compounds, as well as carbon (C) fixation, while suppressing cellulose/hemicellulose decomposition; and (3) soil nutrient cycling processes involving nitrogen (N), phosphorus (P), and sulfur (S) were reprogrammed through changes in both gene abundance (e.g., nifH, pqqC, aprA) and nutrient availability (e.g., NO3-, P). Moreover, specific microbial taxa showed strong correlations with these functional shifts, i.e., Bacteroidota correlated with P metabolism in roots/soil, Actinobacteria and Firmicutes with organic C turnover, and Gemmatimonadetes and Nitrospirae with nitrate cycling dynamics.

**Discussion:**

By integrating poplar’s roles as both a model species and a driver of ecological change, this study elucidates how afforestation shapes soil ecosystems through complex plant-microbe-environment interactions. These findings provide critical insights for sustainable land management strategies.

## 1 Introduction

Poplars (*Populus* spp.) are among the fastest-growing tree species (Tuskan et al., 2006), widely cultivated for timber, biofuels, and ecosystem services such as land rehabilitation, climate change mitigation, windbreaks, and erosion control across mid-latitude plains (Song et al., 2020b). Although poplars can grow well on marginal lands (Laureysens et al., 2005; Guo et al., 2023), their growth and development depends on the functional microbial communities inhabiting the soil–root interface (Beckers et al., 2017; Song et al., 2020a). Much of the research on poplars, however, has been focused on above-ground or subsurface traits—such as growth, photosynthetic capacity, or C storage—with limited connections to belowground nutrient dynamics (Wu et al., 2020). This has affected our understanding on the influences of poplars on soil cycling through plant–microbiome–soil interactions and therefore the sustainability of poplar plantations.

Central to this interaction is the rhizosphere, a dynamic region surrounding plant roots characterized by intense microbial activity (Berg and Smalla, 2009). Roots release substantial amounts of organic compounds derived from photosynthesis and other metabolic processes (Haichar et al., 2014). These compounds are essential and therefore influence the microbial community composition (Sasse et al., 2018). In turn, microbes provide feedback mechanisms affecting the root development and overall plant growth (Pernilla Brinkman et al., 2010)—for example, endophytes are the unique fungi capable of colonizing internal plant tissues, temporarily or permanently, stimulating plant growth and enhancing their natural resistance to biotic and abiotic stresses (Chen et al., 2020). This “rhizosphere effect” intensifies over time (Sasse et al., 2018), associated with changes in root exudate composition and soil physicochemical properties, microbial community succession, and nutrient acquisition and cycling efficiency (Beckers et al., 2017)—for example, younger plantations commonly harbor microbial taxa associated with high nitrogen (N) demand, whereas older plantations tend to favor communities that mobilize phosphorus (P)—reflecting a shift from N to P limitation over time (LeBauer and Treseder, 2008; Zhou et al., 2015). However, the mechanisms underlying these age-related microbial transitions and their collective impacts on C, N, and P cycling across different niches of rhizosphere, roots, and bulk soil remain significant knowledge gaps.

Rhizosphere soil microbiomes have been extensively studied in tree plantations (Banerjee et al., 2019; Romdhane et al., 2022), including poplars (Wu et al., 2025). However, few studies integrate multi-age chronosequences across plant–soil interface niches to capture successional microbial dynamics. Furthermore, limited data link functional shifts in microbial gene abundance with concurrent changes in soil nutrient fluxes. In this study, we analyzed microbial community structures and metabolic functions using metagenomic sequencing technique, along with soil chemical analyses, to investigate microbial–nutrient dynamics across different niches (rhizosphere, roots, and bulk soil) and plantation ages (years 1, 5, and 11 post-planting). Our goal was to understand microbial communities, nutrient contents, and nutrient cycling across different ages of poplar plantations.

## 2 Materials and methods

### 2.1 Experimental site

The study was conducted in a poplar plantation (*Populus* × canadensis ‘I-72/58’) at Fuyu Forest Farm, Qiqihar, China (47°50′ N, 124°47′ E), with a uniform row spacing of 4 × 4 m. The site has an annual mean temperature of 3.4°C, a frost-free period of 125 days, annual mean precipitation ranging from 420 to 480 mm, and annual evaporation of approximately 1,600 to 1,718 mm. The study was conducted in a poplar plantation (*Populus* × canadensis ‘I-72/58’) established in 2011 at unplowed grassland. The soil is classified as Haplic Chernozems (WRB classification) with sandy loam texture (sand 62%/silt 28%/clay 10%). Site preparation involved mechanical clearing followed by pit planting (60 × 60 × 60 cm) of 2-year-root, 1-year-stem transplant seedlings (mean height: 2.0 ± 0.3 m; root collar diameter: 1.5 ± 0.2 cm), with annual manual weeding during years 1–3 but no fertilization applied. Year-1 stands averaged 3.5 ± 0.4 m in height with DBH 3.8 ± 0.7 cm; year-5 reached 12.8 ± 1.2 m in height and 10.3 ± 1.1 cm DBH; year-11 attained 18.6 ± 1.8 m height and 16.7 ± 1.5 cm DBH.

Prior to planting (year 0), the baseline soil characteristics at the experimental site were assessed using composite samples collected from a depth of 0–20 cm. Analyses were performed following standard protocols: soil pH was measured using a glass electrode (soil/water ratio of 1:2.5), EC with a conductivity meter (1:5 soil/water), and moisture content by the gravimetric method. The soil was slightly alkaline (pH = 7.1 ± 0.2, mean ± standard deviation [SD]), with low salinity (EC: 0.25 ± 0.05 dS/m) and moderate moisture content (11.5% ± 1.2%). Nutrient analysis indicated total phosphorus 0.28 ± 0.03 g/kg, available phosphorus (Olsen-P) 20.1 ± 2.4 mg/kg, organic carbon 14.15 ± 1.03 g/kg, ammonium nitrogen 1.02 ± 0.11 mg/kg, nitrate nitrogen 18.23 ± 1.85 mg/kg, and total nitrogen 1.40 ± 0.12 g/kg. Three ages of plantations were selected, years 1, 5, and 11 post-planting, with three plots from each age class (totaling nine plots). Each plot was about 40 m × 30 m to ensure adequate space for sampling across the root–soil interface. All plots were irrigated twice annually (during the dry seasons of each study year: July and August 2022 for 1-year-old stands, 2018–2022 for 5-year-old stands, and 2012–2022 for 11-year-old stands) to prevent soil drought.

### 2.2 Sample collection

The soil and plant samples were collected between August 15 and 20, 2022 (mean daily temperature: 25°C ± 3°C). Samples were collected systematically in plots of different ages. Non-rhizosphere soil samples were collected from a depth of 0–20 cm at locations approximately 50 cm from the base of poplar stems using a sterilized stainless steel auger (inner diameter: 35 mm). Large root fragments (>2 mm) were manually removed, and the remaining soil samples were homogenized using a 2-mm sieve. The roots were excavated carefully using ethanol-sterilized spades to preserve the integrity of root–soil interfaces. ​Bulk soil (defined as soil not adhering to roots after gentle shaking)​​ was removed by shaking the roots three times to ensure separation from rhizosphere soil. Rhizosphere soil (within 0–2 mm of root surfaces) was brushed off using sterile soft-bristle nylon brushes (Bel-Art, USA). Fine root segments (apical 10 cm) were excised using sterile scalpels, rinsed in ice-cold phosphate-buffered saline (PBS, pH 7.0), and immediately flash-frozen in liquid nitrogen. All collected samples were transported and stored at −80°C within 4 h of collection. To prevent cross-contamination, the sampling tools were flame-sterilized using 70% ethanol and a Bunsen burner between each sample.

### 2.3 Nutrient analyses

The concentrations of ammonium (NH_4_^+^), nitrate (NO_3_^-^), total N, total C, total P, and organic C were quantified for both non-rhizosphere and rhizosphere soil samples. NH_4_^+^ and NO_3_^-^ were extracted with 2 M KCl at a soil-to-extractant ratio of 1:5, followed by shaking for 60 min at 250 rpm and 25°C. The extracts were filtered through double-layer quantitative filter paper (Whatman, China) and analyzed using a CleverChem ONE spectrophotometer (Alliance, France) as described by You et al. (2022). Total N, total C, and total P were determined using a stable isotope ratio mass spectrometer (Thermo Fisher Scientific, Germany) following the method by You et al. (2022). The soil samples were air-dried and sieved through a 0.15-mm mesh, and 2–3.5-mg aliquots were encapsulated in tin foil cups and compacted into small pellets for analysis. Organic C was measured using an elemental analyzer (Thermo Fisher Scientific, Germany) after the removal of inorganic C. Furthermore, 5 g of soil was then placed in a conical flask and treated with 20 mL of 6 mol/L HCl for 48 h. The acidified samples were repeatedly rinsed with deionized water until pH 7 was reached, and these were dried at 50°C. The dried samples were packed in tin foil cups and analyzed for organic C content with the elemental analyzer (Thermo Fisher Scientific, Germany).

### 2.4 Metagenomic sequencing

#### 2.4.1 Extraction and detection of genomic DNA

Genomic DNA from soil and root samples was extracted using the Tiangen magnetic bead kit (Omega Bio-tek, Norcross, GA, USA). DNA purity and integrity were evaluated by 1% agarose gel electrophoresis (AGE). DNA concentration was quantified using a Qubit^®^ 2.0 Fluorometer with the appropriate assay kit (Life Technologies, CA, USA). The OD value of the DNA samples was adjusted with sterile water to a range of 1.8–2.0.

The extracted DNA was fragmented to an average size of approximately 400 bp using a Covaris M220 instrument (Gene Company Limited, China) for paired-end library construction. Libraries were prepared using the NEXTFLEX Rapid DNA-Seq Kit (Bioo Scientific, Austin, TX, USA), and adapters containing the full complement of sequencing primer hybridization sites were ligated to the blunt-ended DNA fragments. Paired-end sequencing was performed on an Illumina NovaSeq platform (Illumina Inc., San Diego, CA, USA) at Majorbio Bio-Pharm Technology Co., Ltd. (Shanghai, China) using NovaSeq Reagent Kits according to the manufacturer’s instructions (www.illumina.com).

#### 2.4.2 Sequence quality control and genome assembly

Raw sequencing data were processed using the Majorbio Cloud Platform (www.majorbio.com). Briefly, paired-end Illumina reads were trimmed to remove adapters and low-quality sequences (length <50 bp, quality score <20, or containing N bases) using fastp (Chen et al., 2018) (https://github.com/OpenGene/fastp, version 0.20.0).

Metagenomic assembly was performed using MEGAHIT (Li et al., 2015) (https://github.com/voutcn/megahit, version 1.1.2), which utilizes succinct de Bruijn graphs. Contigs with a length of ≥300 bp were retained as the final assembly and used for subsequent gene prediction and annotation.

#### 2.4.3 Gene prediction, taxonomy, and functional annotation

Gene prediction, taxonomy, and functional annotation were performed on metagenomic sequences derived from microbial communities. Open reading frames (ORFs) were predicted from assembled contigs using MetaGene (Noguchi et al., 2006) (https://github.com/hyattpd/Prodigal). Predicted ORFs ≥100 bp in length were extracted and translated into amino acid sequences based on the NCBI translation table (http://www.ncbi.nlm.nih.gov/Taxonomy/taxonomyhome.html/index.cgi?chapter=tgencodes#SG1).

A non-redundant gene catalog was generated using CD-HIT (Fu et al., 2012) (http://www.bioinformatics.org/cd-hit/, version 4.6.1) with thresholds of 90% sequence identity and 90% coverage. High-quality reads were then aligned to the non-redundant gene catalog to calculate gene abundance using SOAPaligner (Li et al., 2008) (https://github.com/ShujiaHuang/SOAPaligner, version 2.21), with 95% identity threshold.

Representative sequences from the non-redundant gene catalog were aligned to the NCBI NR database using Diamond (Buchfink et al., 2015) (https://github.com/bbuchfink/diamond, version 0.8.35), with an e-value cutoff of 1e^-5^ for taxonomic annotations. Cluster of Orthologous Groups (COG) annotation was performed by aligning representative sequences against the eggNOG database using Diamond with the same e-value threshold. KEGG functional annotation was conducted by aligning sequences to the Kyoto Encyclopedia of Genes and Genomes (KEGG) database (http://www.genome.jp/kegg/) using Diamond, also with an e-value cutoff of 1e^-5^. Functional genes involved in the C, N, P, and sulfur (S) cycles were identified through KEGG pathway annotation, and changes in their relative abundance were determined. Annotation of carbohydrate-active enzymes was conducted using hmmscan (http://bcb.unl.edu/dbCAN2/download/Databases/) against the CAZy database (http://www.cazy.org/) with an e-value cutoff of 1e^-5^. All sequence data generated in this project have been deposited in the NCBI database under accession number SRP445747.

### 2.5 Data analysis

A two-way factorial ANOVA was conducted to evaluate the main and interactive effects of plantation age (years 1, 5, and 11) and niche (non-rhizosphere, rhizosphere, root) on nutrient-cycling-related variables (NH_4_^+^, NO_3_^-^, total N, total C, total P, and organic C). Analyses were performed using IBM SPSS Statistics 26.0 (Chicago, USA). Tukey’s HSD test (*α* = 0.05) was used for *post hoc* multiple comparisons. Prior to conducting parametric tests, data normality was assessed using the Shapiro–Wilk test (*p* > 0.05 for all variables), and homogeneity of variances was evaluated with Levene’s test (*p* > 0.1).

Alpha diversity quantifies species richness and evenness within individual microbial communities, serving as a fundamental metric in microbial ecology to assess ecosystem health and complexity. In this study, we employed three complementary indices to capture distinct dimensions of diversity.​​ Chao1 index​ estimates total species richness, emphasizing the detection of rare taxa. Shannon index​ combines richness and evenness, weighting toward abundant species. Simpson index​ prioritizes dominant species sensitivity. This multi-index approach mitigates the limitations of single metrics and aligns with microbial ecology best practices.​​ For analysis, operational taxonomic units (OTUs) were clustered at 97% sequence similarity using USEARCH (v11), and indices were calculated via the estimateR function in vegan (v3.2.0) based on rarefied OTU tables.

This ordination technique transforms species abundance data (Hellinger-transformed OTU tables) into orthogonal principal components (PCs), where PC1 and PC2 capture the maximum variance in community structure. The analysis was implemented via vegan (v3.2.0), with OTU abundance vectors as input and plantation age/niche as supplementary variables. The resulting PC axes were interpreted based on species scores (>|0.4| correlation) and vector angles representing environmental correlations. Taxonomic composition at the phylum level was visualized with bar plots generated by the phyloseq package (v1.42.0), excluding OTUs with relative abundances <0.1%. All results are reported as mean ± SD.

## 3 Results

### 3.1 Diversity of microbial communities

**Figure 1:**
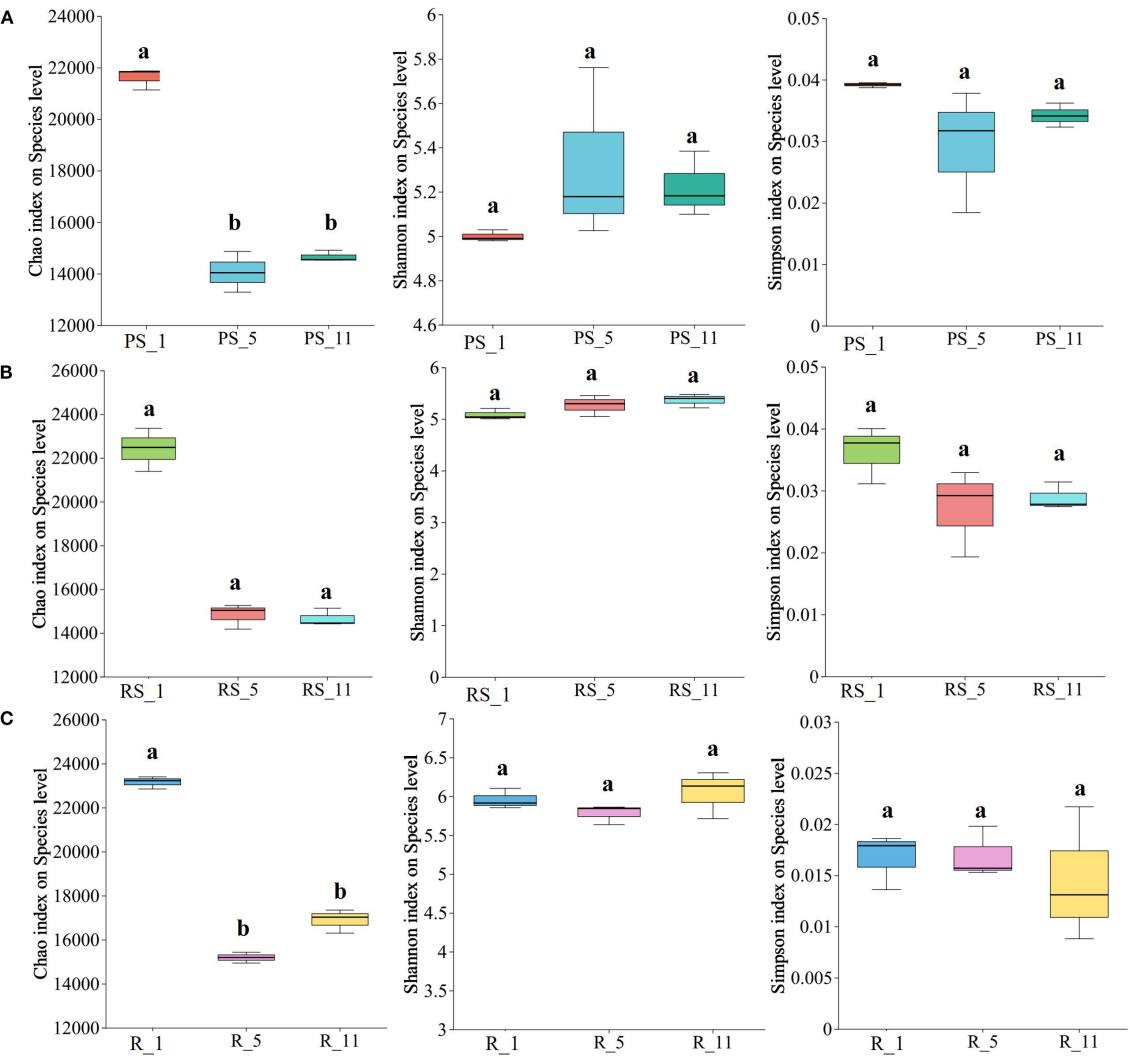
Effects of poplar plantation age (years 1, 5, and 11) on the richness of microbial communities (Chao, Shannon, and Simpson diversity indices) in **(A)** non-rhizosphere soil (PS), **(B)** rhizosphere soil (RS), and **(C)** roots (R). Letters (a, b, c) denote statistically significant differences among ages.

The taxonomic richness of microbial communities is defined as the number of distinct operational taxonomic units (OTUs). The richness of species groups, as measured by the Chao1 index, declined in all three niches (non-rhizosphere soil, rhizosphere soil, and roots) at 5 and 11 years post-planting (*P*-values < 0.05). More pronounced reductions were in the non-rhizosphere soil and root samples (**Figures 1A–C**). Comparatively, the changes of Simpson index and Shannon index with plantation age were not significant (**Figures 1A–C**).

### 3.2 Microbial community composition and characteristics

**Figure 2:**
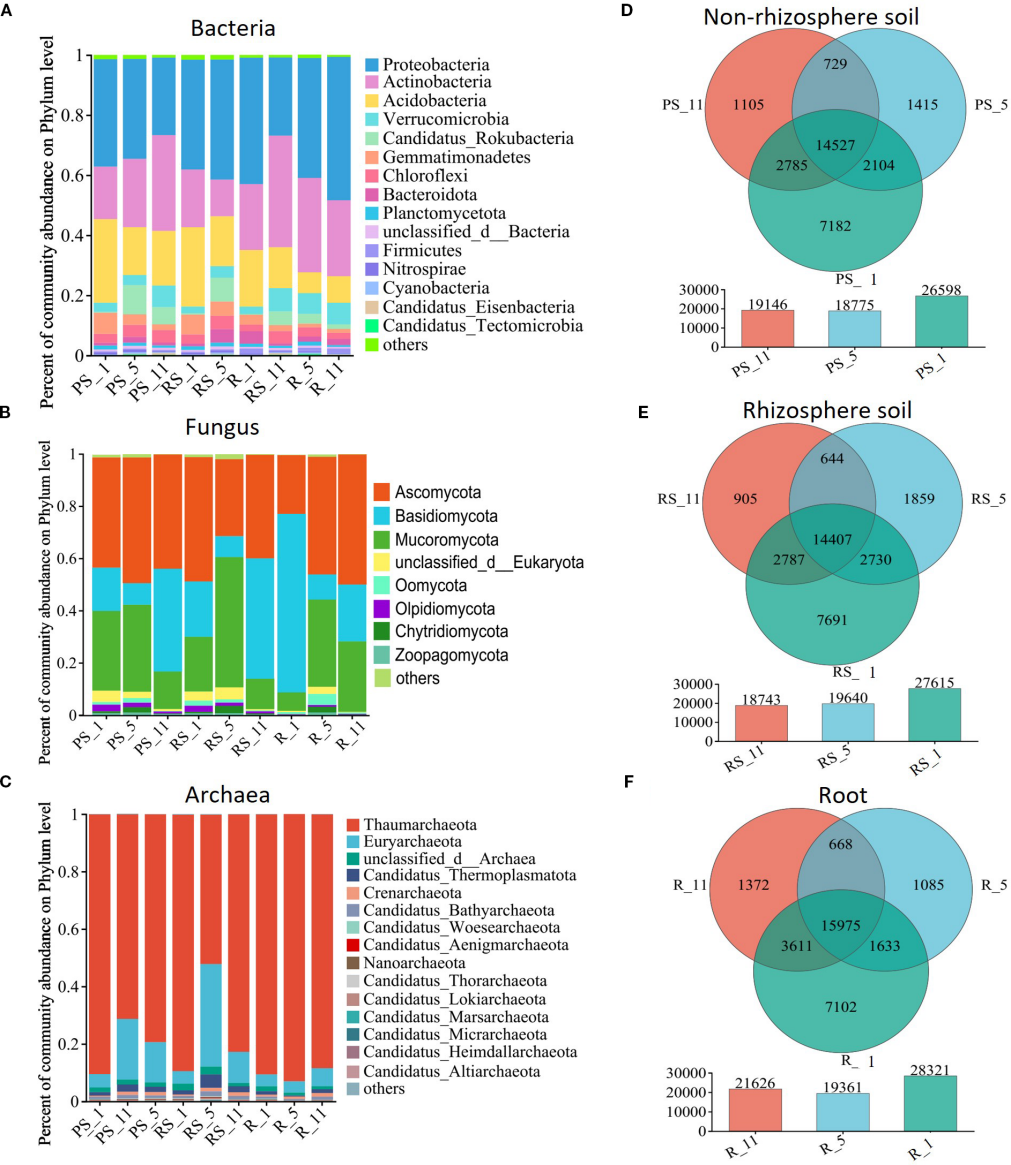
Effects of poplar plantation age (years 1, 5, and 11) on microbial community composition at the phylum level **(A)** bacterial, **(B)** fungal, and **(C)** archaeal communities in non-rhizosphere soil (PS), rhizosphere soil (RS), and roots (R). Venn diagrams illustrate the unique and shared microbial taxa within **(D)** non-rhizosphere soil, **(E)** rhizosphere soil, and **(F)** roots.

The core bacterial phyla identified in all three niches were Proteobacteria, Actinobacteria, Acidobacteria, Verrucomicrobia, and Candidatus_Rokubacteria, collectively accounting for over 80% of the bacterial community (**Figure 2A**). The abundance of these phyla shifted with plantation age and niche. In non-rhizosphere soil, the composition of Proteobacteria, Gemmatimonadetes, and Planctomycetes decreased with plantation age (*P*-value <0.01), whereas Actinobacteria, Verrucomicrobia, and Chloroflexi abundances increased with plantation age (**Figure 2A** and **Supplementary Table S5**) (*P*-value <0.01). In rhizosphere soil, the composition of Proteobacteria, Candidatus_Rokubacteria, and Chloroflexi peaked at year 5 (**Figure 2A** and **Supplementary Table S6**) (*P*-value <0.01), whereas Actinobacteria reached the lowest at year 5, and Acidobacteria and Verrucomicrobia increased with plantation age (*P*-value <0.01). The composition of Bacteroidetes peaked at year 5 in both non-rhizosphere and rhizosphere soils (*P*-value <0.05). In roots, Actinobacteria and Nitrospirae peaked and Acidobacteria, Verrucomicrobia, and Bacteroidota reached the lowest at year 5. The Bacteroidetes increase occurred primarily at year 1 post-planting (*P*-value <0.05) (**Figure 2A** and **Supplementary Table S7**).

The core fungal phyla were Ascomycota, Basidiomycota, and Mucoromycota (>85%) and exhibited niche-specific post-planting dynamics (**Figure 2B**, **Supplementary Tables S5–S7**). In non-rhizosphere soils, the composition of Ascomycota and Mucoromycota peaked at year 5 (*P*-value <0.01), while that of Ascomycota and Basidiomycota in rhizosphere soil decreased from years 1 to 5 before increasing at year 11, contrasting with the patterns of rise and decline of Mucoromycota (*P*-value <0.05). In roots, the composition of Basidiomycota decreased along with the increase of Mucoromycota and Ascomycota composition with age (*P*-value <0.05).

The core Archaeal (Thaumarchaeota, Euryarchaeota, and Candidatus_Thermoplasmatota, >90%) showed different temporal patterns. Thaumarchaeota composition decreased with compensatory Euryarchaeota expansion in non-rhizosphere and rhizosphere soils from years 1 to 5 (*P*-value <0.01). Microbial richness progressively declined with plantation age in all niches, although the roots showed partial recovery at year 11 post-planting (**Figures 2D–F**).

### 3.3 Co-occurrence networks of microbiomes

**Figure 3:**
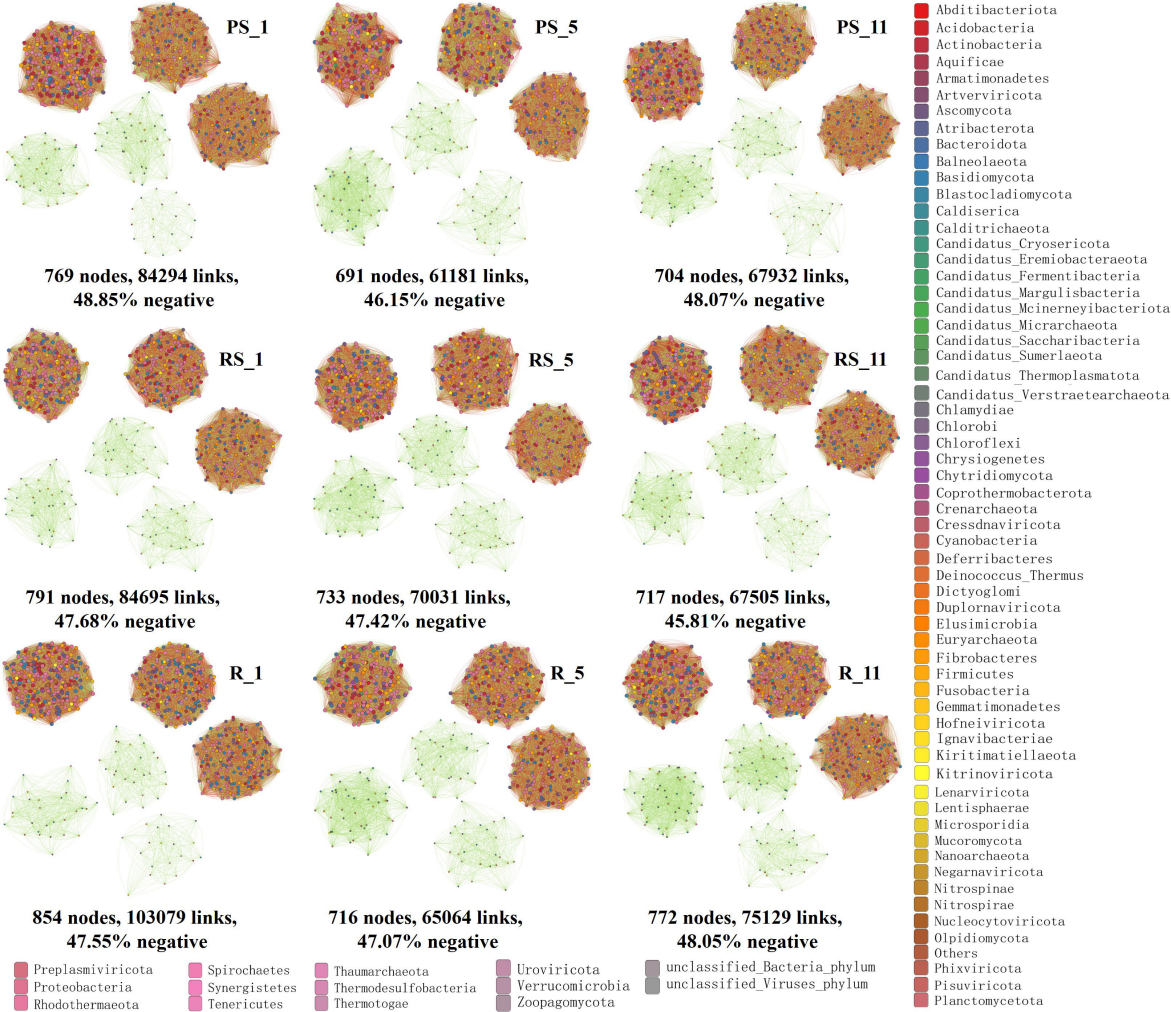
Co-occurrence networks of microbial communities in non-rhizosphere soil (PS), rhizosphere soil (RS), and root (R) at years 1, 5, and 11 post-planting.

Microbial co-occurrence networks were progressively simplified with plantation age in all niches (**Figure 3**, **Supplementary Tables S1–S4**). The key topological parameters—including total nodes, links, and average degree—declined at years 5 and 11 post-planting. Root networks demonstrated increased modularity and negative correlation ratios at year 11. Bacterial, archaeal, and fungal communities showed a higher network complexity at year 1 compared to years 5 and 11 post-planting (**Supplementary Figures S1–S3**).

### 3.4 Impact of poplar cultivation on the gene abundance of nutrient cycling

**Figure 4:**
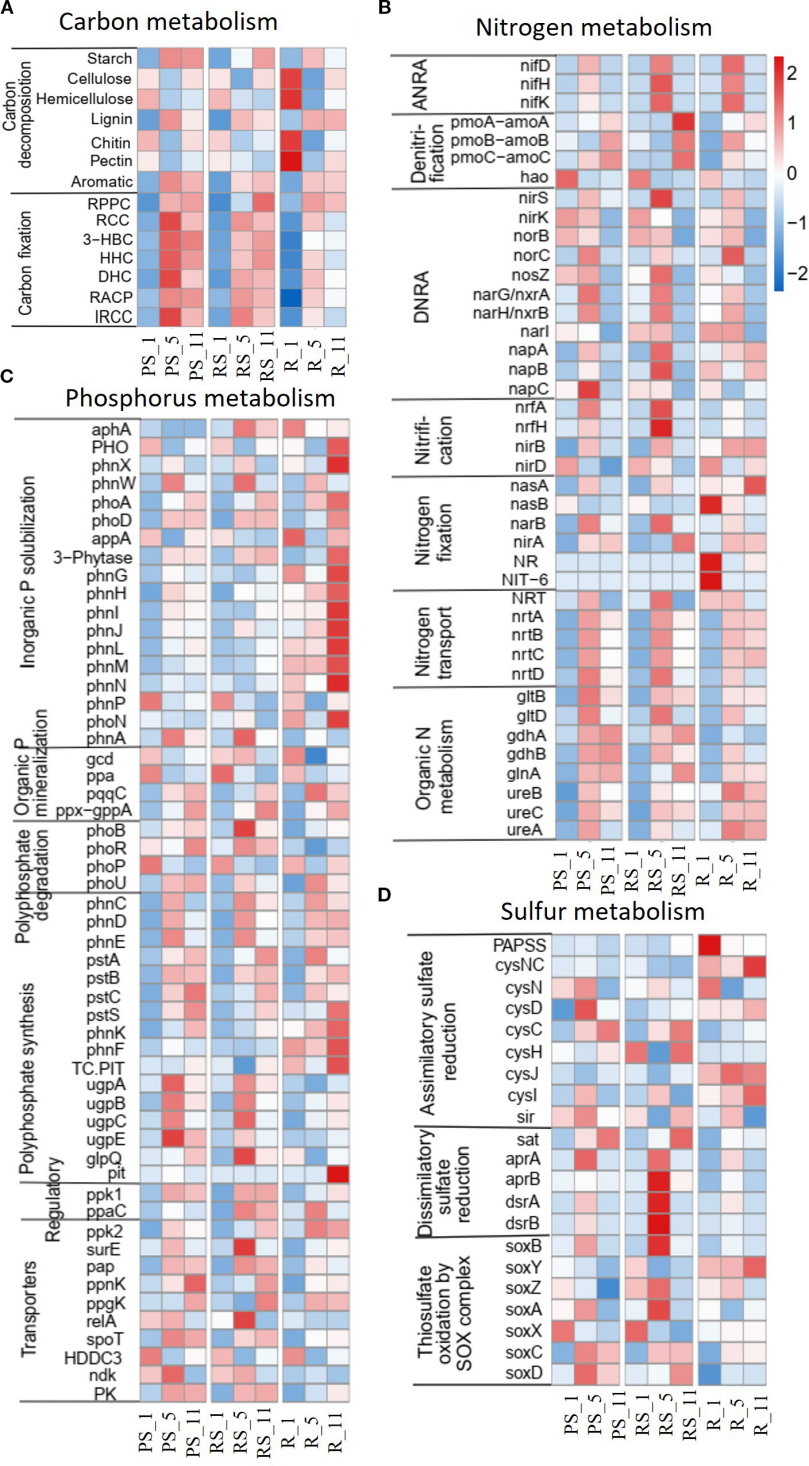
Effects of poplar plantation age (years 1, 5, and 11) on the abundance of genes related to major biogeochemical cycles in three niches, **(A)** C cycle, **(B)** N cycle, **(C)** P cycle, and **(D)** S cycle. The key C fixation pathways in panel **(A)** include RPPC (reductive pentose phosphate cycle; Calvin cycle), RCC (reductive citrate cycle; Arnon–Buchanan cycle), 3-HBC (3-hydroxypropionate bi-cycle), HHC (hydroxypropionate–hydroxybutyrate cycle), DHC (dicarboxylate–hydroxybutyrate cycle), RACP (reductive acetyl-CoA pathway; Wood–Ljungdahl pathway), and IRCC (incomplete reductive citrate cycle). **(B)** DNRA refers to dissimilatory nitrate reduction to ammonium, while ANRA is assimilatory nitrate reduction to ammonium. The other letters in (b–d) represent gene names.

**Figure 5:**
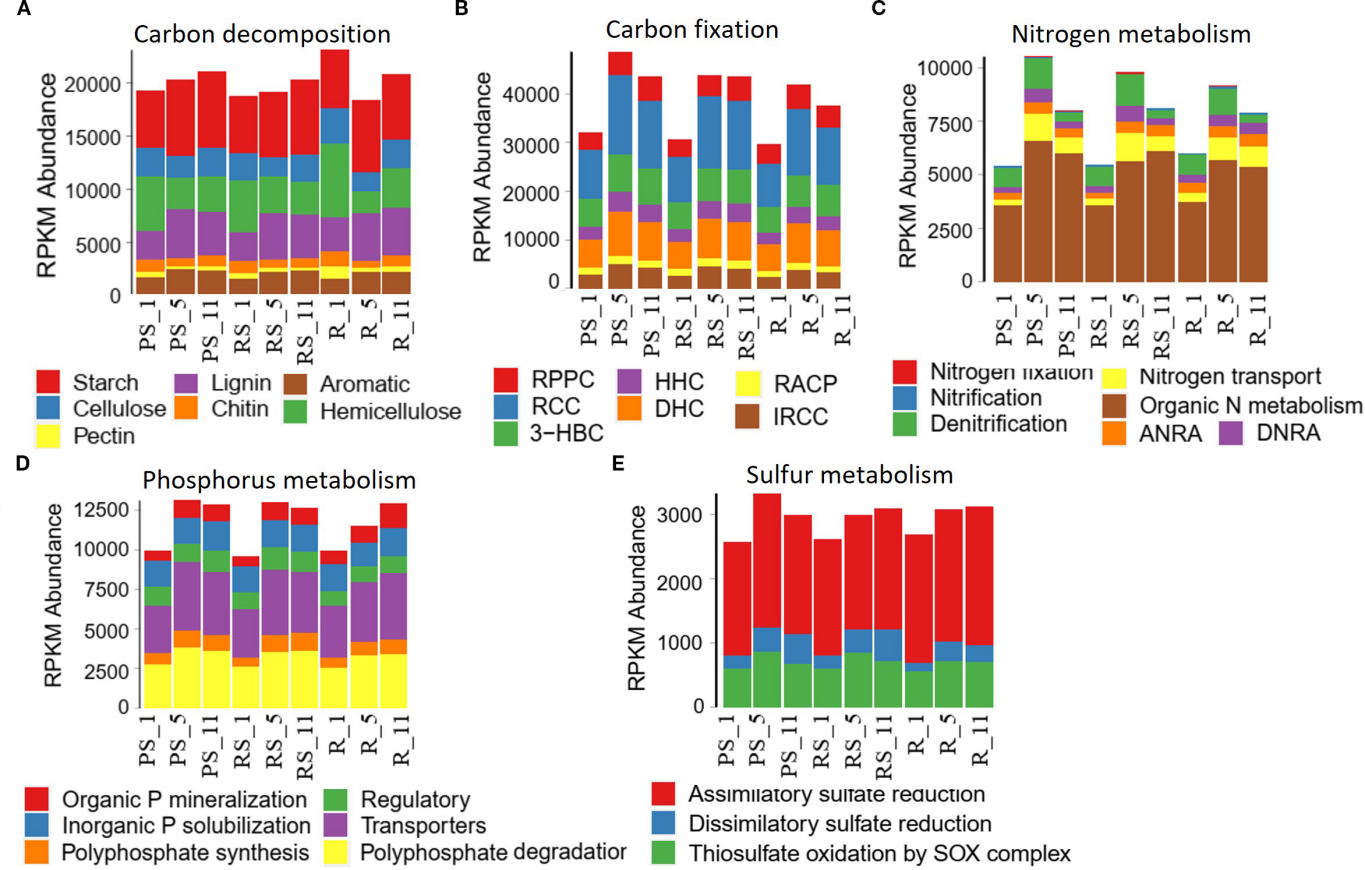
Effects of poplar plantation age (years 1, 5, and 11) on total gene abundance related to nutrient cycling processes, **(A)** C decomposition, **(B)** C fixation, **(C)** N cycling, **(D)** P cycling, and **(E)** S cycling. The abbreviations in panel **(B)** represent key C fixation pathways: RPPC (reductive pentose phosphate cycle; Calvin cycle), RCC (reductive citrate cycle; Arnon–Buchanan cycle), 3-HBC (3-hydroxypropionate bi-cycle), HHC (hydroxypropionate–hydroxybutyrate cycle), DHC (dicarboxylate–hydroxybutyrate cycle), RACP (reductive acetyl-CoA pathway; Wood–Ljungdahl pathway), and IRCC (incomplete reductive citrate cycle).

Poplar cultivation altered the abundance of nutrient cycling genes in all niches. Gene abundance refers to the normalized count of metagenomic sequencing reads mapped to specific functional genes, calculated as fragments per kilobase per million mapped reads (FPKM) to correct for gene length and sequencing depth bias. In carbon cycling, the gene abundance with starch, lignin, aromatic compound metabolism, and C fixation increased, while that with cellulose/hemicellulose metabolisms decreased with plantation age, with the maximal changes at year 5 post-planting (**Figures 4A**, **5A, B**). Temporal shifts in nitrogen cycling gene abundance emerged under poplar cultivation, characterized by the coordinated upregulation of genes mediating ammonium retention pathways. Assimilatory nitrate reduction (*nasA*, *narB*, *nirA*), nitrogen fixation (*nifD/H/K*), and dissimilatory nitrate reduction to ammonium (*nrfA/H*, *nirB*) increased after 5 years, while denitrification genes exhibited divergent temporal patterns—transient increases at year 5 (nirK) but persistent decreases (*nirD*). Concurrently, genes facilitating N transport and organic N metabolism increased consistently across stand development, collectively demonstrating a microbial strategy prioritizing nitrogen conservation over gaseous loss pathways (**Figures 4B**, **5C**). In phosphorus cycling, the gene abundance with inorganic P solubilization was relatively stable, while that with organic P mineralization, polyphosphate degradation/synthesis, and phosphate transport increased after planting (**Figures 4C**, **5D**). In sulfur cycling, the gene abundance with dissimilatory reduction and SOX-mediated thiosulfate oxidation increased, while assimilatory reduction did not change significantly after planting (**Figure 5E**).

**Figure 6:**
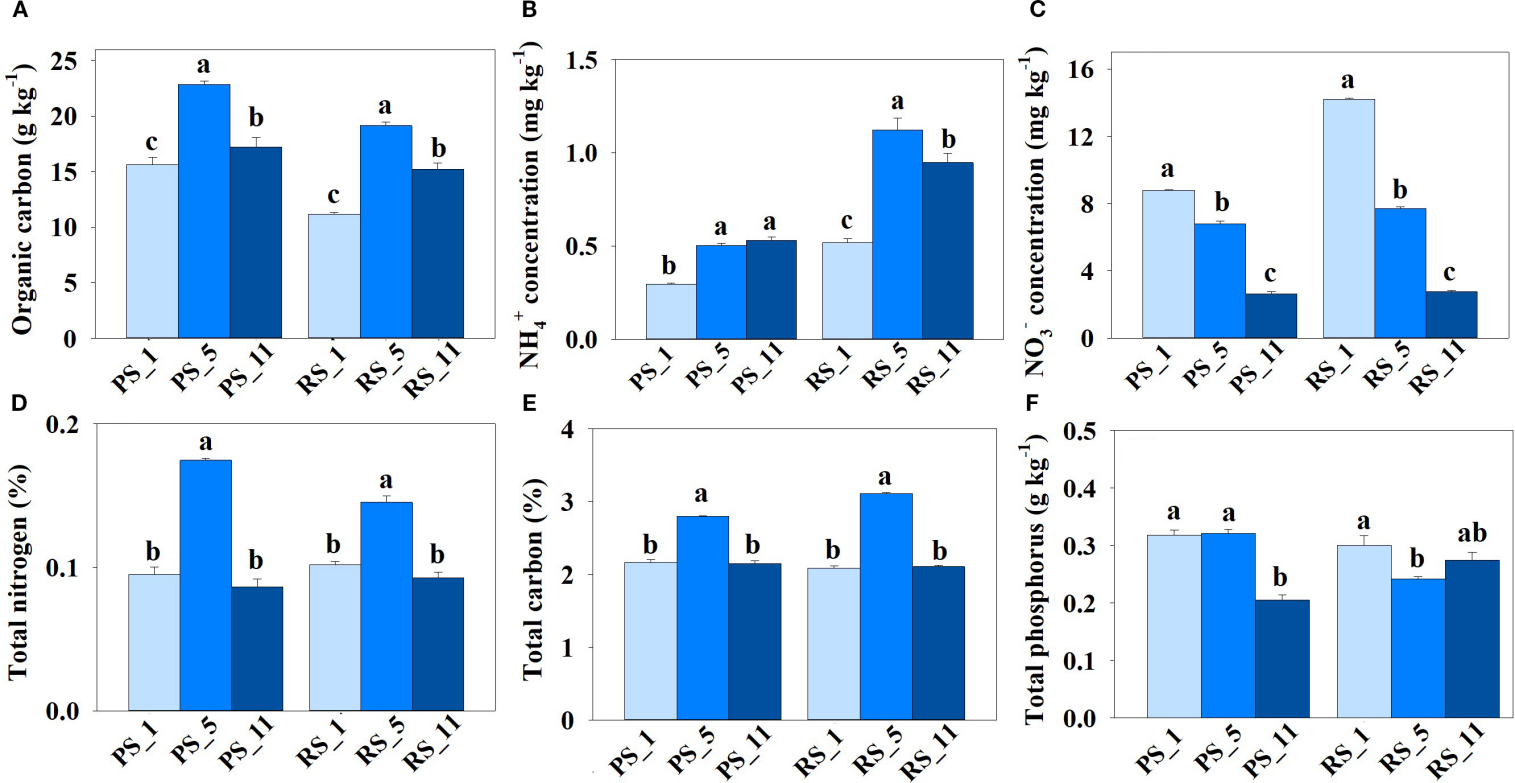
Impact of poplar plantation age (years 1, 5, and 11) on nutrient concentrations, **(A)** organic C, **(B)** NH_4_^+^, **(C)** NO_3_^-^, **(D)** total N, **(E)** total C, and **(F)** total P. Lowercase letters (a, b, c) indicate significant differences between non-rhizosphere and rhizosphere soils.

Along with the changes of gene abundance were the increase of soil organic carbon and NH_4_^+^ concentrations and decrease of NO_3_^−^ concentration (**Figures 6A–C**). Total carbon and nitrogen in non-rhizosphere and rhizosphere soils started to increase at year 5, whereas total phosphorus decreased either from year 11 in non-rhizosphere soil or year 5 in rhizosphere soil (**Figures 6D–F**).

### 3.5 Roles of microorganisms in nutrient cycling

**Figure 7:**
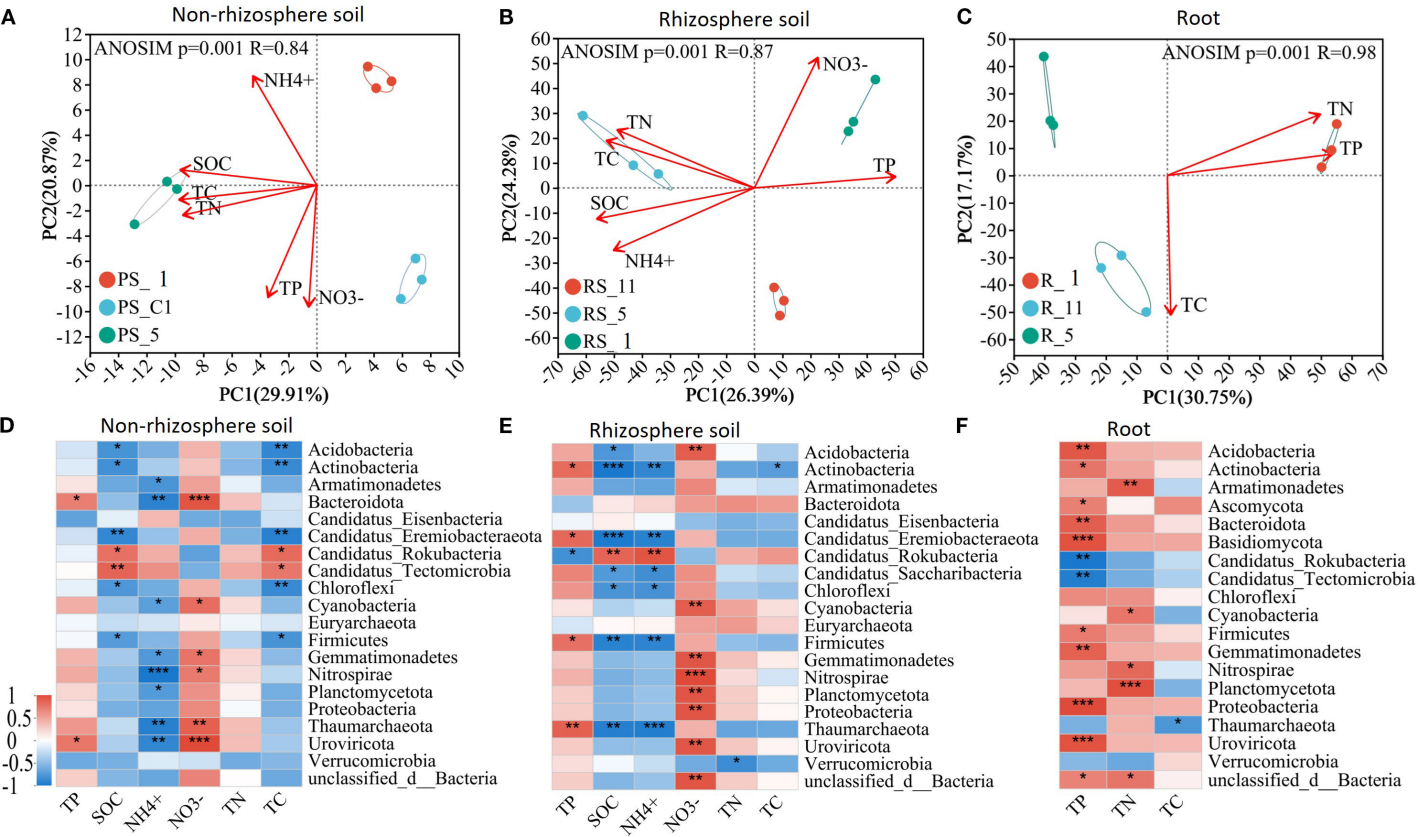
Changes of microbial community structure in **(A)** non-rhizosphere soil, **(B)** rhizosphere soil, and **(C)** roots and of correlations between microbial communities and nutrient concentrations in **(D)** non-rhizosphere soil, **(E)** rhizosphere soil, and **(F)** roots with poplar plantation age (years 1, 5, and 11).

Poplar cultivation shifted microbial community structures (**Figures 7A–C**). Organic C, NH_4_^+^, NO_3_^−^, total N, total C, and total P correlated with community composition in non-rhizosphere and rhizosphere soils, while total N, total C, and total P correlated with root microbiota (**Figures 7A–C**). In P dynamics, Bacteroidota positively correlated with total P in non-rhizosphere soil and roots, while Actinobacteria and Firmicutes showed niche-divergent P responses. In C cycling, Acidobacteria and Actinobacteria were inversely linked to organic C pools. In N transformations, Nitrospirae and Gemmatimonadetes were consistently associated with NO_3_^−^ accumulation (**figures 7D–F**).

**Figure 8:**
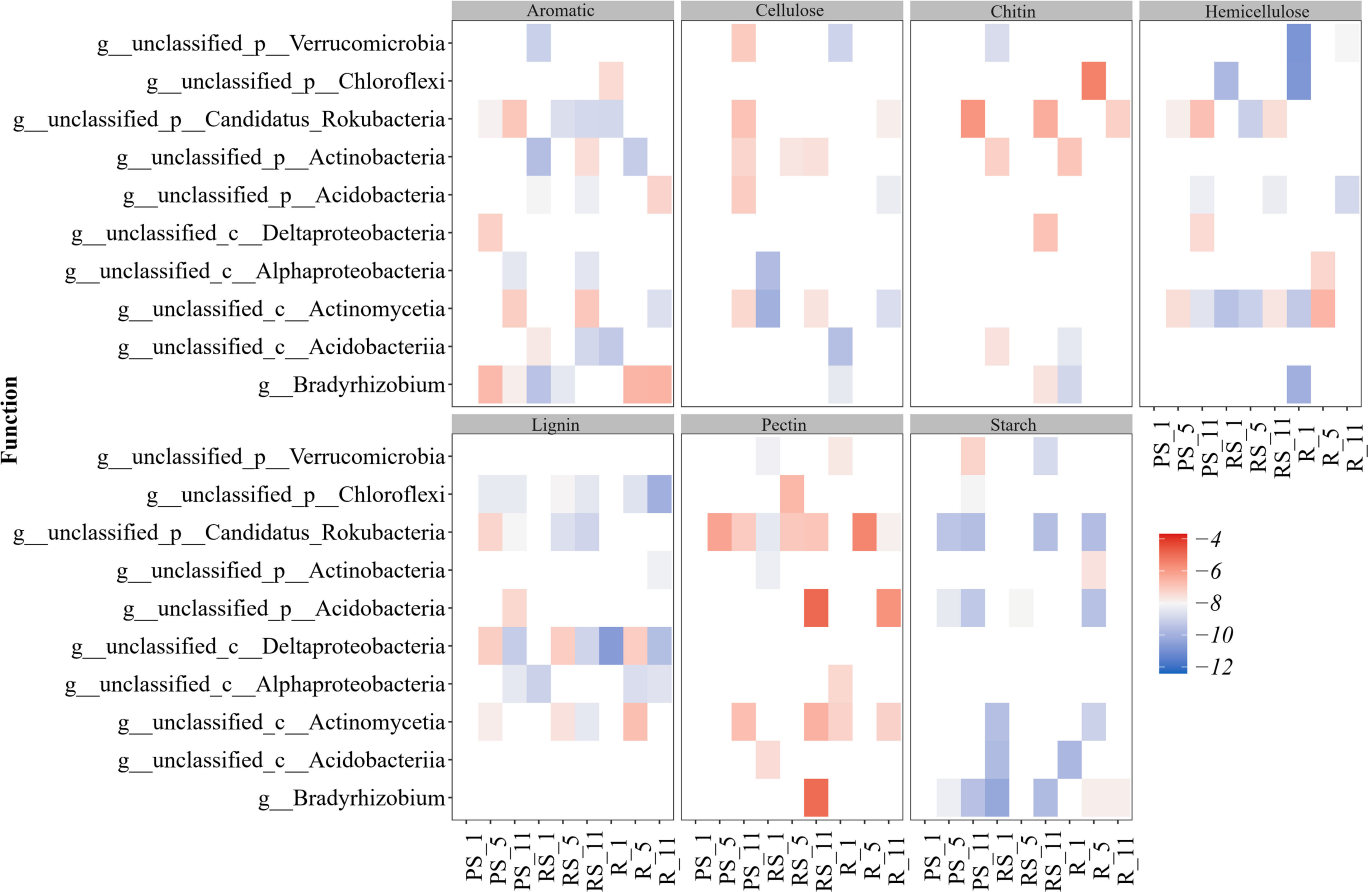
Contribution of microbial taxa to C decomposition processes under different poplar plantation ages (years 1, 5, and 11).

Correlation analysis revealed genus-level metabolic specialization and niche-optimized nutrient cycling (**Figure 8**, **Supplementary Figures S4–S7**). Bradyrhizobium dominated aromatic metabolism, Verrucomicrobia taxa mediated multiple C fixation pathways, and Chloroflexi/Rokubacteria influenced nitrification processes.

## 4 Discussion

### 4.1 Effects of poplar cultivation on microbial communities

Our integrated analysis reveals that poplar plantations drive a niche-optimized microbial succession strategy, fundamentally restructuring community composition and function to balance metabolic efficiency with enhanced resource acquisition (Cregger et al., 2018). This restructuring manifests in three interconnected core shifts with significant implications for nutrient cycling and plantation sustainability. Fist, the contrasting post-planting dynamics among three diversity indices of this study indicate management-induced microbial specialization (Cregger et al., 2018) that manifests as simplified microbial co-occurrence networks (Banerjee et al., 2019). This post-planting change resembles the patterns observed in managed *Eucalyptus* plantations (Romdhane et al., 2022), suggesting that agroforestry practices prioritize core metabolic functions at the expense of interactive complexity (de Vries et al., 2018). This microbial specialization may reflect a trade-off between functional efficiency and ecosystem resilience. Second, key microbial taxa exhibit niche-driven dynamics in this study. Bacteroidota dominance shifted spatially and temporally (Gan et al., 2021), i.e., enriched in year 1 roots and predominant in year 5 rhizosphere soil. This spatial–temporal progression indicates active root–rhizosphere microbiota recruitment rather than passive displacement. Potential drivers include root exudate gradients and irrigation-induced moisture shifts, though mechanistic validation requires future metabolomics (Zhalnina et al., 2018). However, the groups conventionally linked to soil organic carbon (SOC) stabilization, including Actinobacteria and Firmicutes (Liang et al., 2017), were unexpectedly associated with reduced SOC, possibly due to exudate-induced priming effects (Kaiser et al., 2015). This paradox is explained by root exudate-induced priming effects: low-molecular-weight compounds (e.g., malic acid, glucose) released from poplar roots stimulate microbial activity, accelerating the decomposition of native SOC (Kuzyakov et al., 2000). Third, this study found that the relative suppression of nitrifiers (Nitrospirae) along with enrichment of nitrogen-fixing species suggests a possible trade-off in nitrogen strategies (Gan et al., 2021). This reconfiguration prioritizes biological nitrogen fixation over nitrification-mediated losses, redirecting nitrogen flux toward plant-available forms (NH_4_^+^) while minimizing NO_3_^−^ leaching—a response amplified by poplar root exudates under nitrogen-limited conditions (Richardson and Simpson, 2011). A similar trade-off may have also occurred in fungal dynamics of this study, i.e., enrichment of Mucoromycota (often housing arbuscular mycorrhizal fungi—AMF) in the year 5 rhizosphere soil coincided with the decline of P levels, suggesting compensatory recruitment under P limitation (Sawers et al., 2017). This efficiency–resilience trade-off creates intervention windows ​at diversity bottlenecks (e.g., year 5 post-planting), where resource competition peaks and management legacies compress microbial recovery phases, enabling targeted organic amendments to restore functional redundancy (Allison and Martiny, 2008).

### 4.2 Effects of poplar cultivation on nutrient cycling

Our results show that poplar plantations drive a fundamental metabolic reorganization of microbial communities, shifting their priority from decomposing recalcitrant substrates toward utilizing labile carbon sources (e.g., starch), as suggested by (Cregger et al., 2018). In the carbon cycle, this reorganization manifests as suppressed cellulose metabolism coupled with enhanced starch/lignin degradation capacity (Philippot et al., 2013; Romdhane et al., 2022), aligning with intensively managed *Eucalyptus* systems (Romdhane et al., 2022) but with natural *Populus* forests where cellulose metabolism dominates (Kuzyakov, 2010; Philippot et al., 2013). While the enhanced degradation of recalcitrant carbon (e.g., lignin) and carbon fixation potential may promote long-term soil organic carbon sequestration (Kögel-Knabner, 2017), the suppressed cellulose and hemicellulose decomposition likely slows organic matter turnover (Liang et al., 2017), limiting short-term nutrient availability (Fontaine et al., 2004).

Within the nitrogen cycle, decreased Nitrospirae abundance and upregulated DNRA (dissimilatory nitrate reduction to ammonium) in this study may suggest a core nitrogen conservation strategy, mirroring microbial behavior in fertilized maize systems (Coskun et al., 2017). Notably, the accumulation of soil nitrate facilitated by Gemmatimonadetes (Ma et al., 2020) deviates from their typical function in arid soils, indicating habitat-specific functional remodeling (DeBruyn et al., 2011). This result found that phosphorus acquisition is characterized by a microbial shift from inorganic P solubilization toward efficient organic P mineralization (Richardson and Simpson, 2011). Central to this is that Bacteroidota can leverage its phosphatase capabilities to establish a “root endosphere–rhizosphere” continuum (Gan et al., 2021), progressively upregulating phosphatase genes (Zhalnina et al., 2018) to dramatically enhance P capture efficiency (Sasse et al., 2018).

The functional contributions of key microbial taxa dictate the efficacy of these pathways. For instance, Actinobacteria and Firmicutes, conventionally linked to SOC stabilization (Liang et al., 2017), show an unexpected association with carbon loss with our study, revealing the risk of exudate-induced priming effects (Kaiser et al., 2015). Concurrently, the enrichment of diazotrophs alongside the relative suppression of Nitrospirae (Gan et al., 2021) underscores poplar’s strategic prioritization of biological nitrogen fixation and DNRA for ammonium conservation (Leininger et al., 2006; Prosser and Nicol, 2012). The dominance of Bradyrhizobium in aromatic compound metabolism and the role of Verrucomicrobia in regulating carbon fixation pathways further highlight the niche specificity of microbial functions (Wang et al., 2018).

This research showed that microbial-driven organic P mineralization (Zhalnina et al., 2018) endows poplar systems with superior P utilization efficiency compared to coniferous monocultures (Sasse et al., 2018), directly supporting biomass accumulation. The DNRA-dominated N conservation strategy (Coskun et al., 2017) reduces reactive N loss, but sustained suppression of nitrification (Gan et al., 2021) may constrain nitrate supply, necessitating staged N fertilization to balance demand during peak growth phases. While enhanced lignin degradation aids carbon sequestration (Kögel-Knabner, 2017), short-term nutrient limitation stemming from suppressed cellulose decomposition (Fontaine et al., 2004) requires supplementing labile organic amendments (e.g., green manure) in young stands to maintain growth momentum.

During the mid-rotation P depletion phase (e.g., year 5 post-planting), the inoculation of phosphate-solubilizing probiotics or AM fungi can enhance P acquisition (Sawers et al., 2017). Simultaneously, engineering the rhizosphere soil environment to modulate root exudate composition (e.g., boosting organic acid secretion) can selectively activate beneficial microbial functions (Berendsen et al., 2012; Pii et al., 2015; Wang et al., 2015), synergistically enhancing nutrient cycling efficiency and plantation productivity.

## 5 Conclusions

Our study shows that poplar cultivation substantially reshaped microbial community composition (bacteria, fungi, archaea) and functionally altered nutrient cycling through clearly defined taxon–function relationships. Specifically, reduced complexity in microbial co-occurrence networks correlated with suppressed cellulose and hemicellulose decomposition, likely reflecting the disruption of key decomposer consortia critical for plant-derived C turnover. The enriched microbial genes related to starch and lignin degradation, as well as C fixation pathways, indicate shifts driven by root-exudate-derived C sources. Functional reconfiguration of N metabolism—with reduced denitrification linked to *Nitrospirae* depletion and increased inorganic N transport associated with *Proteobacteria*—suggests microbial community adaptation to poplar root N demands. Additionally, microbial-driven P solubilization via *Bacteroidota*-mediated phosphatase activity and coupled reductions in organic C mediated by *Actinobacteria* highlight microbiome-level trade-offs inherent in nutrient acquisition strategies. Collectively, these taxon–function relationships elucidate how poplar-driven microbial shifts directly influence C/N/P cycling efficiencies. Consequently, we propose two actionable strategies: implement early-stage fertilization (<5 years) to mitigate N/P decline coupled with late-stage (<11 years) mycorrhizal management to sustain organic P mineralization while preserving keystone taxa like N-fixing species and rare Acidobacteria to enhance nutrient cycling resilience. Such targeted microbiome stewardship must integrate process-centered adaptive silviculture frameworks, harmonizing soil biodiversity conservation with productivity enhancement through science-guided microbial interventions.

## Data Availability

The datasets presented in this study can be found in online repositories. The names of the repository/repositories and accession number(s) can be found in the article/**Supplementary Material**.
