# Supplementary material for: Integrated metagenomic and soil chemical analyses revealed shifts of microbial nutrient cycling with poplar plantation age
Source: Front Plant Sci. 2025 Oct 20;16:1513281. doi: 10.3389/fpls.2025.1513281 (PMC12580573; doi:10.3389/fpls.2025.1513281)
Supplement: Supplementary file 1 [file DataSheet1.docx]

Supplementary Material

# Supplementary Figures and Tables

## Supplementary Figures


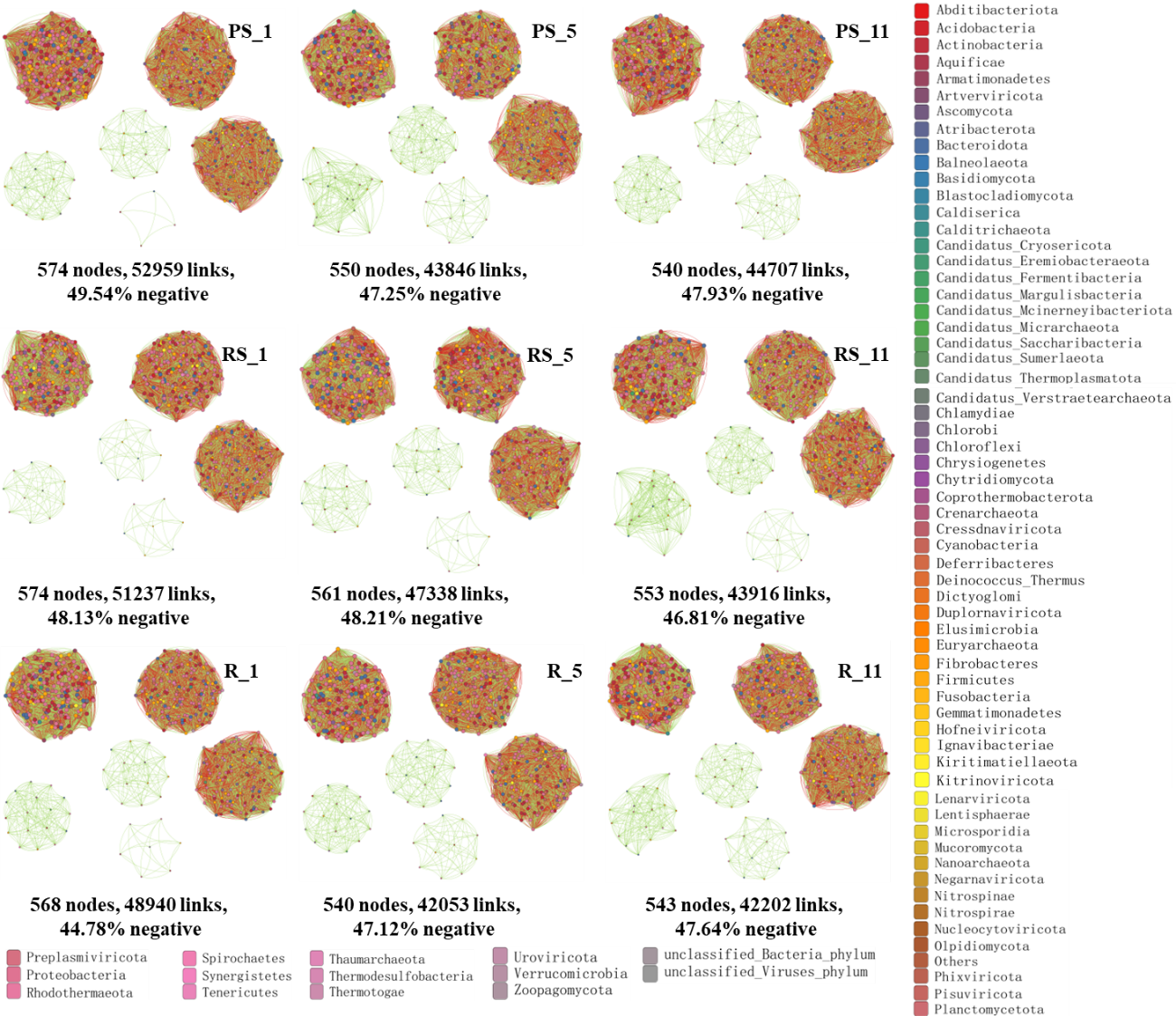


**Supplementary Figure** 1 Co-occurrence network of bacteria in non-rhizosphere soil, rhizosphere soil and roots. The three treatments were 1-year-old poplars (PS_1/RS_1/R_1), 5-year-old poplars (PS_5 /RS_5 /R_5), and 11-year-old poplars (PS_11 /RS_11/R_11).


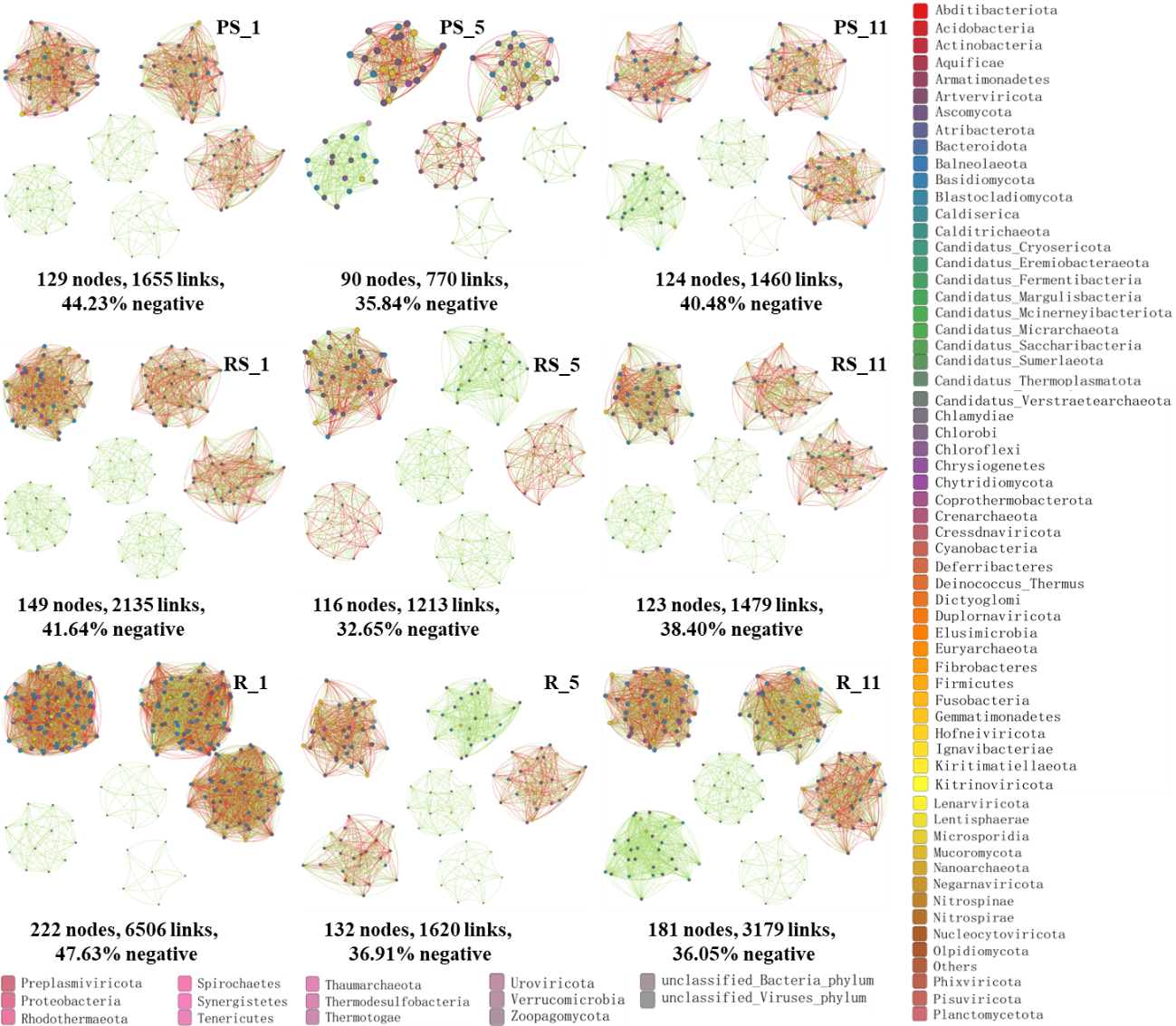


**Supplementary Figure** 2 Co-occurrence network of fungus in non-rhizosphere soil, rhizosphere soil and roots. The three treatments were 1-year-old poplars (PS_1/RS_1/R_1), 5-year-old poplars (PS_5 /RS_5 /R_5), and 11-year-old poplars (PS_11 /RS_11/R_11).


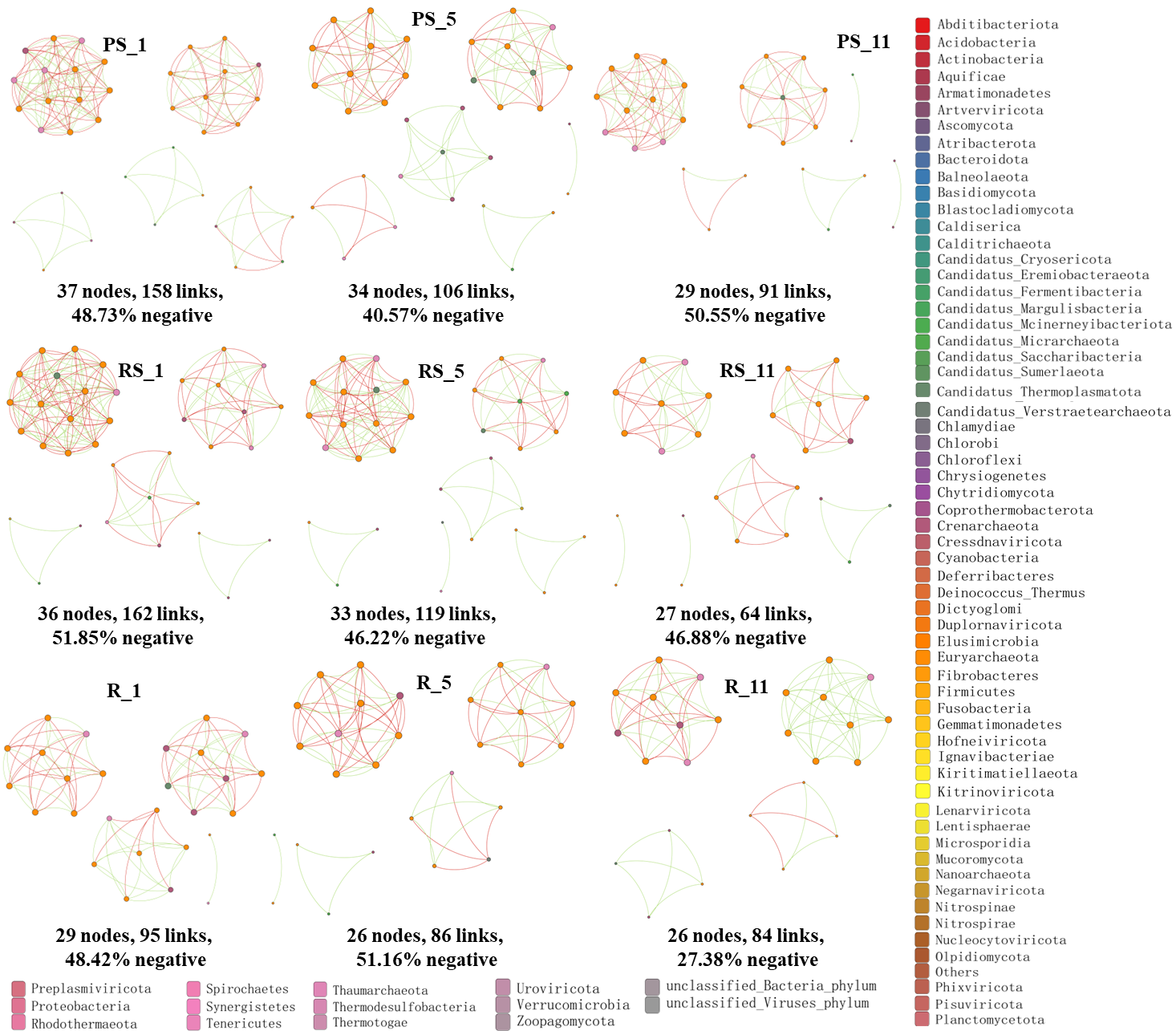


**Supplementary Figure** 3 Co-occurrence network of archaea in non-rhizosphere soil, rhizosphere soil and roots. The three treatments were 1-year-old poplars (PS_1/RS_1/R_1), 5-year-old poplars (PS_5 /RS_5 /R_5), and 11-year-old poplars (PS_11 /RS_11/R_11).


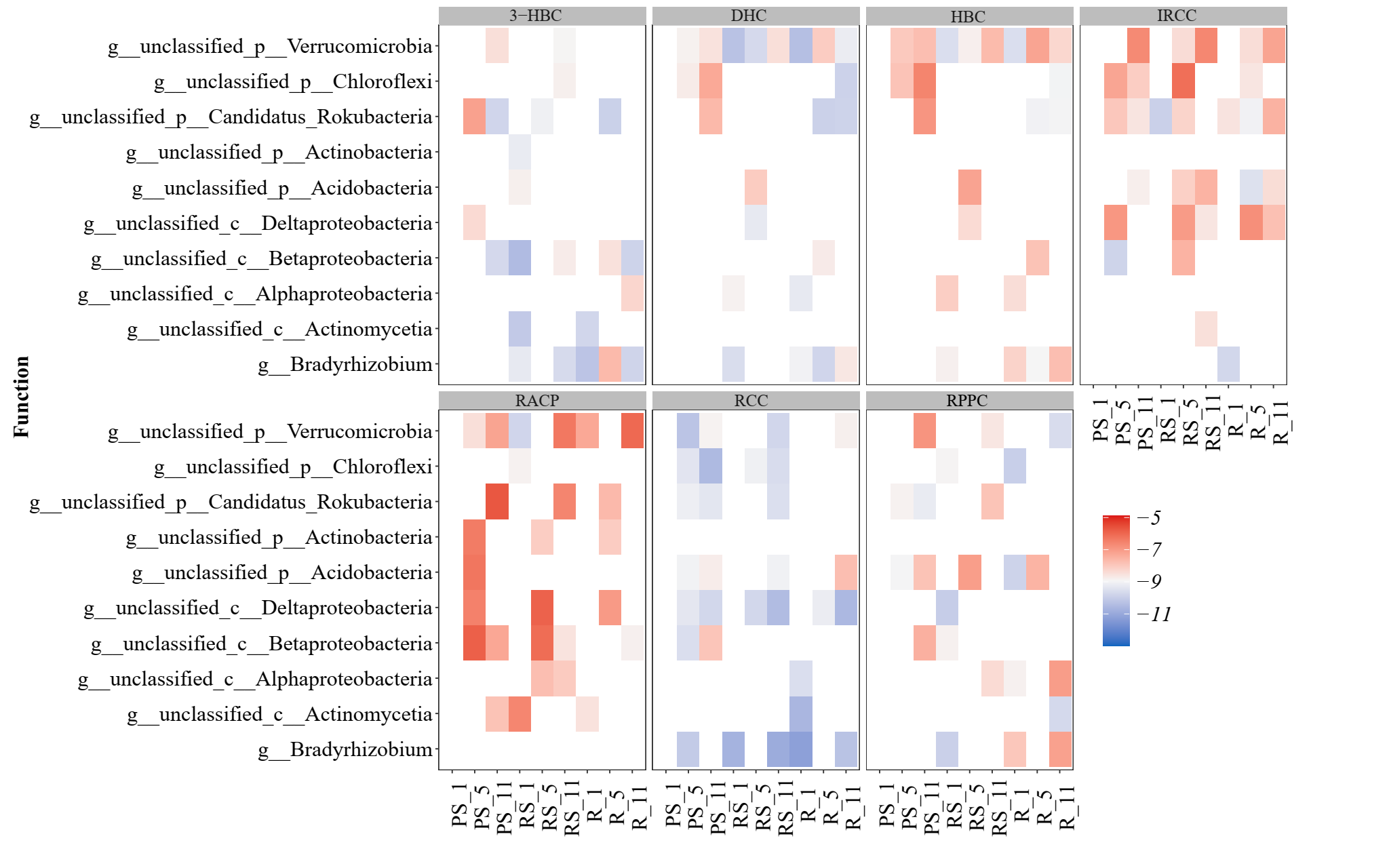


**Supplementary Figure** 4 The contribution of microorganisms to carbon fixation. The three treatments were 1-year-old poplars (PS_1/RS_1/R_1), 5-year-old poplars (PS_5 /RS_5 /R_5), and 11-year-old poplars (PS_11 /RS_11/R_11).


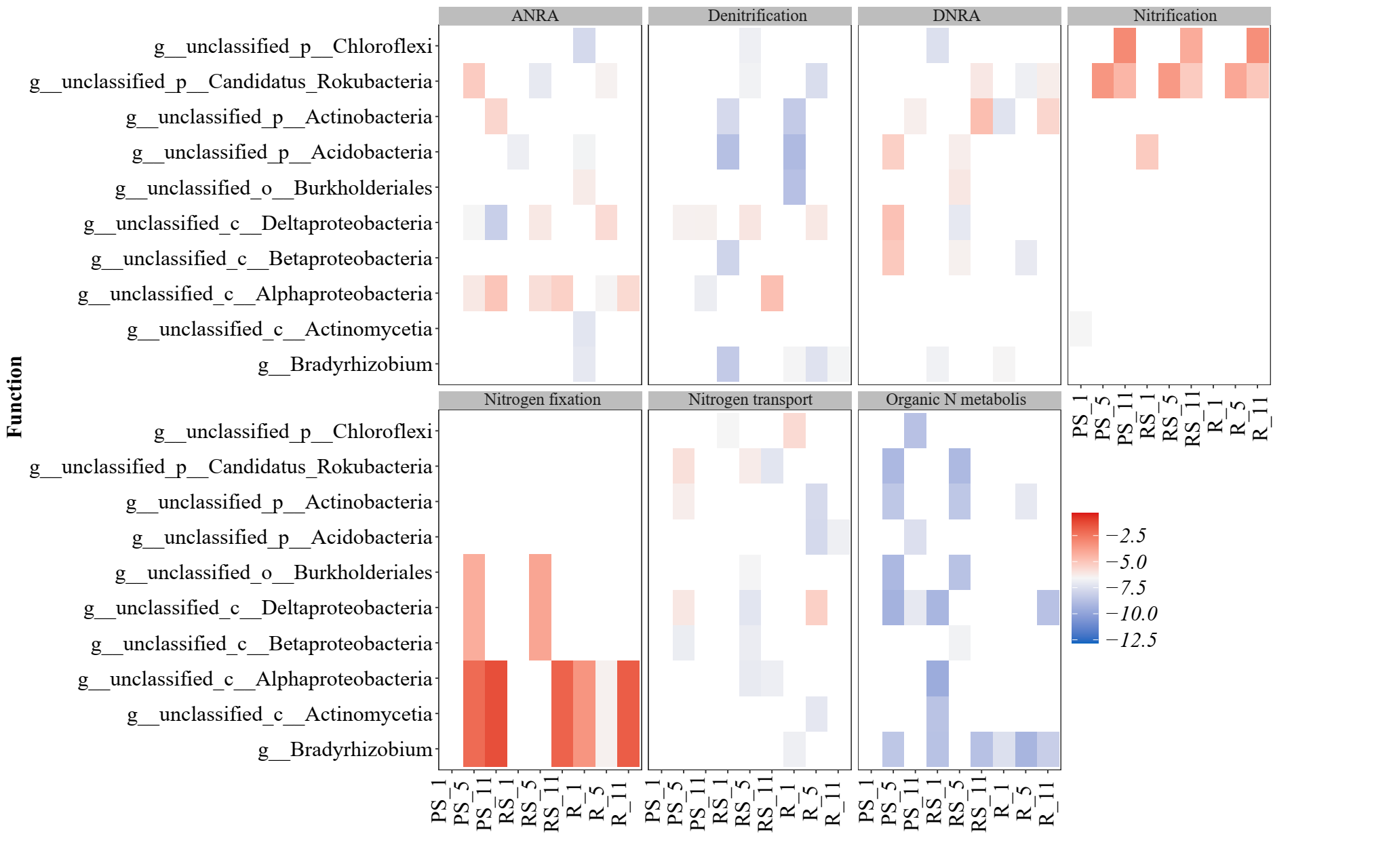


**Supplementary Figure** 5 The contribution of microorganisms to N cycle. The three treatments were 1-year-old poplars (PS_1/RS_1/R_1), 5-year-old poplars (PS_5 /RS_5 /R_5), and 11-year-old poplars (PS_11 /RS_11/R_11).


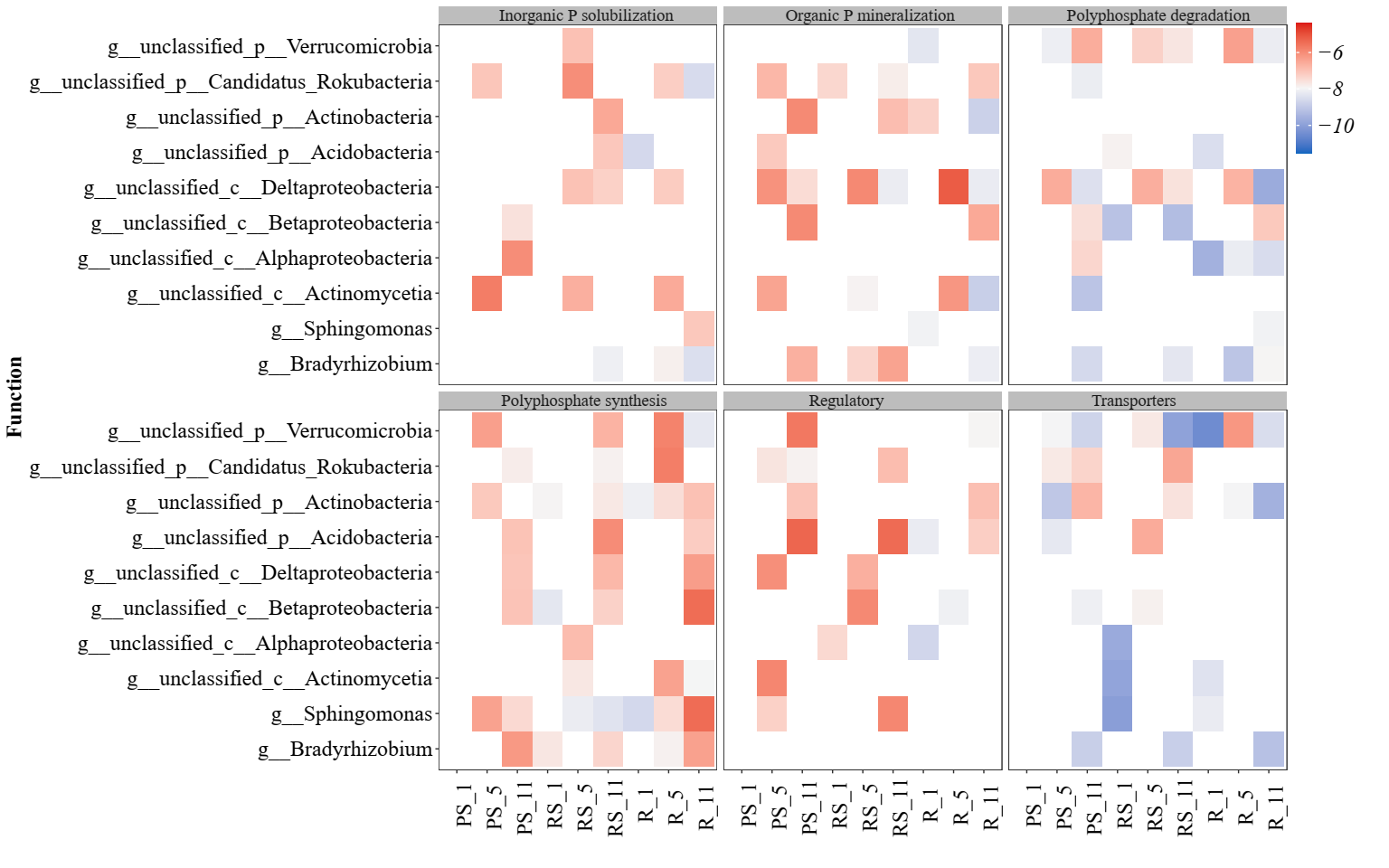


**Supplementary Figure** 6 The contribution of microorganisms to P cycle. The three treatments were 1-year-old poplars (PS_1/RS_1/R_1), 5-year-old poplars (PS_5 /RS_5 /R_5), and 11-year-old poplars (PS_11 /RS_11/R_11).


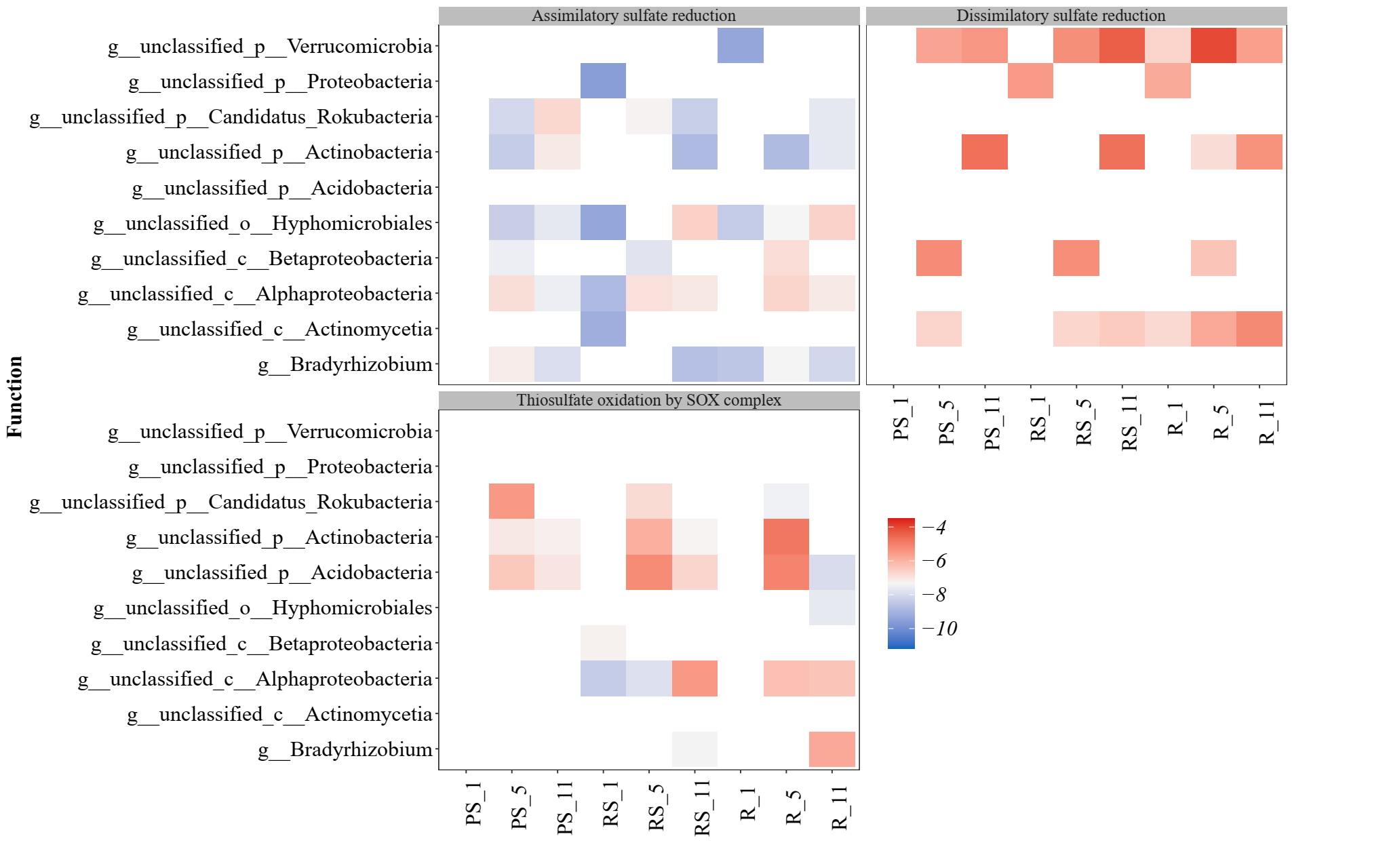


**Supplementary Figure** 7 The contribution of microorganisms to S cycle. The three treatments were 1-year-old poplars (PS_1/RS_1/R_1), 5-year-old poplars (PS_5 /RS_5 /R_5), and 11-year-old poplars (PS_11 /RS_11/R_11).

## Supplementary table

**Supplementary** **Table** 1 Network topological parameters of microbial communities

| Group | Total nodes | Total links | Average degree (avgK) | Average clustering coefficient (avgCC) | Average path distance (GD) | Modularity |
| --- | --- | --- | --- | --- | --- | --- |
| PS_1 | 769 | 84294 | 219.23 | 1 | 1 | 0.59 |
| PS_5 | 691 | 61181 | 177.08 | 1 | 1 | 0.67 |
| PS_11 | 704 | 67932 | 192.99 | 1 | 1 | 0.61 |
| RS_1 | 791 | 84695 | 214.15 | 1 | 1 | 0.65 |
| RS_5 | 733 | 70031 | 191.08 | 1 | 1 | 0.65 |
| RS_11 | 717 | 67505 | 188.30 | 1 | 1 | 0.65 |
| R_1 | 854 | 103079 | 241.40 | 1 | 1 | 0.65 |
| R_5 | 716 | 65064 | 181.74 | 1 | 1 | 0.67 |
| R_11 | 772 | 75129 | 194.63 | 1 | 1 | 0.67 |

**Supplementary** **Table** 2 Network topological parameters of bacterial communities

| Group | Total nodes | Total links | Average degree (avgK) | Average clustering coefficient (avgCC) | Average path distance (GD) | Modularity |
| --- | --- | --- | --- | --- | --- | --- |
| PS_1 | 574 | 52959 | 184.53 | 1 | 1 | 0.56 |
| PS_5 | 550 | 43846 | 159.44 | 1 | 1 | 0.64 |
| PS_11 | 540 | 44707 | 165.58 | 1 | 1 | 0.58 |
| RS_1 | 574 | 51237 | 178.53 | 1 | 1 | 0.63 |
| RS_5 | 561 | 47338 | 168.76 | 1 | 1 | 0.65 |
| RS_11 | 553 | 43916 | 158.83 | 1 | 1 | 0.65 |
| R_1 | 568 | 48940 | 172.32 | 1 | 1 | 0.61 |
| R_5 | 540 | 42053 | 155.75 | 1 | 1 | 0.65 |
| R_11 | 543 | 42202 | 155.44 | 1 | 1 | 0.65 |

**Supplementary** **Table** 3 Network topological parameters of fungus communities

| Group | Total nodes | Total links | Average degree (avgK) | Average clustering coefficient (avgCC) | Average path distance (GD) | Modularity |
| --- | --- | --- | --- | --- | --- | --- |
| PS_1 | 129 | 1655 | 25.66 | 1 | 1 | 0.71 |
| PS_5 | 90 | 770 | 17.11 | 1 | 1 | 0.73 |
| PS_11 | 124 | 1460 | 23.55 | 1 | 1 | 0.77 |
| RS_1 | 149 | 2135 | 28.66 | 1 | 1 | 0.71 |
| RS_5 | 116 | 1213 | 20.91 | 1 | 1 | 0.72 |
| RS_11 | 123 | 1479 | 24.05 | 1 | 1 | 0.71 |
| R_1 | 222 | 6506 | 58.61 | 1 | 1 | 0.66 |
| R_5 | 132 | 1620 | 24.55 | 1 | 1 | 0.75 |
| R_11 | 181 | 3179 | 35.13 | 1 | 1 | 0.75 |

**Supplementary** **Table** **4** Network topological parameters of archaea communities

| Group | Total nodes | Total links | Average degree (avgK) | Average clustering coefficient (avgCC) | Average path distance (GD) | Modularity |
| --- | --- | --- | --- | --- | --- | --- |
| PS_1 | 37 | 158 | 8.54 | 1 | 1 | 0.58 |
| PS_5 | 34 | 106 | 6.24 | 1 | 1 | 0.68 |
| PS_11 | 29 | 91 | 6.28 | 1 | 1 | 0.54 |
| RS_1 | 36 | 162 | 9 | 1 | 1 | 0.51 |
| RS_5 | 33 | 119 | 7.21 | 1 | 1 | 0.51 |
| RS_11 | 27 | 64 | 4.74 | 1 | 1 | 0.67 |
| R_1 | 29 | 95 | 6.55 | 1 | 1 | 0.66 |
| R_5 | 26 | 86 | 6.62 | 1 | 1 | 0.61 |
| R_11 | 26 | 84 | 6.46 | 1 | 1 | 0.62 |

Supplementary Table 5 Microbial community composition in non-rhizosphere soil at the phylum level

| Phylum | PS_1 | PS_5 | PS_11 | P |
| --- | --- | --- | --- | --- |
| p__Proteobacteria | 614290.72±25586.74 | 663106.59±35882.29 | 514124.47±18007.08 | P<0.01 |
| p__Actinobacteria | 300417.58±33525.94 | 453462.47±165868.76 | 634722.31±94646.94 | P<0.05 |
| p__Acidobacteria | 477860.81±5955.24 | 317283.54±74897.13 | 361679.14±25903.74 | P<0.05 |
| p__Verrucomicrobia | 52650.87±6392.49 | 67055.98±7065.97 | 143452.9±33268.67 | P<0.01 |
| p__Candidatus_Rokubacteria | 4840.8±1201.4 | 193768.71±105957.35 | 113816.62±34984.61 | P<0.05 |
| p__Gemmatimonadetes | 120621.91±9006.81 | 70150.49±24869.49 | 38821.6±1581.91 | P<0.01 |
| p__Chloroflexi | 50661.7±1733.48 | 79611.22±14714.5 | 81544.61±3601.71 | P<0.01 |
| p__Bacteroidota | 15606.89±2309.85 | 37910.3±3233.65 | 18779.62±4047.34 | P<0.001 |
| p__Planctomycetota | 23307.36±528.21 | 21168.04±988.85 | 15760.67±1345.09 | P<0.001 |
| p__unclassified_d__Bacteria | 10841.56±464.5 | 18951.25±453.96 | 14614.24±2497.38 | P<0.01 |
| p__Firmicutes | 4886.91±112.66 | 7330.36±961.88 | 6371.08±479.04 | P<0.01 |
| p__Nitrospirae | 12420.01±3719.78 | 15625.16±6908.27 | 7208.82±1360.63 | P>0.05 |
| p__Cyanobacteria | 4556.53±86.62 | 11340.06±2646.61 | 6675.01±975.52 | P<0.01 |
| p__Candidatus_Eisenbacteria | 752.05±40.14 | 2979.72±1731.47 | 12324.93±5522.39 | P<0.05 |
| p__Candidatus_Tectomicrobia | 243.96±84.62 | 7226.35±847.21 | 3622.28±699.99 | P<0.001 |
| p__Ascomycota | 511.65±35.72 | 894.63±42.58 | 1782.18±66.94 | P<0.001 |
| p__Basidiomycota | 200.45±48.28 | 154.61±89.84 | 1603.25±553.55 | P<0.01 |
| p__Mucoromycota | 370.34±57.32 | 619.7±90.39 | 584.81±103.79 | P<0.05 |
| p__unclassified_d__Eukaryota | 52.32±8.12 | 44.41±21.33 | 30.1±6.88 | P>0.05 |
| p__Oomycota | 12.36±5.2 | 32.68±46.41 | 8.88±3.4 | P>0.05 |
| p__Olpidiomycota | 31.15±6.28 | 32.26±14.88 | 27.75±2.7 | P>0.05 |
| p__Chytridiomycota | 8.38±1.23 | 38.34±32.69 | 9.85±1.09 | P>0.05 |
| p__Zoopagomycota | 10.24±2.42 | 19.8±2.87 | 21.27±11.35 | P>0.05 |
| p__Thaumarchaeota | 21975.5±694.65 | 20581.06±3239.75 | 9921.21±3371.33 | P<0.01 |
| p__Euryarchaeota | 1104.4±359.47 | 3632.76±277.44 | 2965.26±685.54 | P<0.01 |
| p__unclassified_d__Archaea | 424.67±30.89 | 395.84±68.6 | 229.44±79.19 | P<0.05 |
| p__Candidatus_Thermoplasmatota | 294.79±48.9 | 475.95±186.21 | 351.17±39.46 | P>0.05 |
| p__Crenarchaeota | 94.12±20.71 | 304.66±128.58 | 157.28±81.39 | P>0.05 |
| p__Candidatus_Bathyarchaeota | 172.44±4.54 | 236.84±29.93 | 166.79±10.15 | P<0.01 |
| p__Candidatus_Woesearchaeota | 76.89±6.41 | 91.9±3.66 | 50.98±4.9 | P<0.001 |
| p__Candidatus_Aenigmarchaeota | 34.86±5.57 | 45.13±11.79 | 31.09±6.81 | P>0.05 |
| p__Nanoarchaeota | 31.06±3.3 | 59.68±40.61 | 11.13±11.36 | P>0.05 |
| p__Candidatus_Thorarchaeota | 18.56±3 | 25.74±18.99 | 9.73±3.57 | P>0.05 |
| p__Candidatus_Lokiarchaeota | 13.47±3.94 | 13.15±9.67 | 9.63±6.68 | P>0.05 |
| p__Candidatus_Marsarchaeota | 12.6±4.86 | 14.3±7.96 | 11.39±6.1 | P>0.05 |
| p__Candidatus_Micrarchaeota | 11.67±2.82 | 11.09±3.02 | 2.12±3.67 | P<0.05 |
| p__Candidatus_Heimdallarchaeota | 5.57±0.75 | 19.76±7.08 | 7.08±2.02a | P<0.05 |
| p__Candidatus_Altiarchaeota | 10.76±2.43 | 22.29±26.24 | 10.22±11.34 | P>0.05 |

Supplementary Table 6 Microbial community composition in rhizosphere soil at the phylum level

| Phylum | RS_1 | RS_5 | RS_11 | P |
| --- | --- | --- | --- | --- |
| p__Proteobacteria | 616547.35±24638.51 | 773340.61±42846.03 | 513486.58±15233.22 | P<0.001 |
| p__Actinobacteria | 323974.18±45445.67 | 236558.13±49941.76 | 732879.55±46550.26 | P<0.001 |
| p__Acidobacteria | 445302.6±32740.67 | 321110.27±88853.78 | 268769.43±10331.13 | P<0.05 |
| p__Verrucomicrobia | 37167.72±11476.98 | 74714.59±15693.73 | 152288.14±25498.86 | P<0.01 |
| p__Candidatus_Rokubacteria | 7212.25±4293.5 | 152990.28±47089.07 | 90026.17±27193.79 | P<0.01 |
| p__Gemmatimonadetes | 112424.25±15750.5 | 92617.36±14208.27 | 39397.52±4650.01 | P<0.01 |
| p__Chloroflexi | 50974.99±3164.8 | 85804.25±9523.36 | 80492.21±4083.47 | P<0.05 |
| p__Bacteroidota | 14685.55±2463.9 | 86515.49±2452.56 | 15145.72±2565.37 | P<0.05 |
| p__Planctomycetota | 20846.56±882.74 | 24642.16±3065.29 | 15574.04±761.87 | P<0.01 |
| p__unclassified_d__Bacteria | 10293.72±424.16 | 19113.27±1280.61 | 13675.72±716.12 | P<0.001 |
| p__Firmicutes | 4906.1±98.97 | 6101.73±217.24 | 6501.28±861.39 | P<0.05 |
| p__Nitrospirae | 9754.19±2578.91 | 13847.04±5312.47 | 7275.92±2808.3 | P>0.05 |
| p__Cyanobacteria | 4239.81±249.14 | 10566.67±1943.56 | 6516.55±693.68 | P<0.01 |
| p__Candidatus_Eisenbacteria | 1018.06±665.34 | 4127.99±697.93 | 10134.86±5718.76 | P<0.05 |
| p__Candidatus_Tectomicrobia | 352.99±200.92 | 4130.84±1012.92 | 4362.39±1156.35 | P<0.01 |
| p__Ascomycota | 602.61±47.72 | 546.28±140.93 | 1530.46±307.36 | P<0.05 |
| p__Basidiomycota | 268.26±52.05 | 148.89±52.82 | 1776.41±355.25 | P<0.05 |
| p__Mucoromycota | 265.27±7.44 | 924.54±750.98 | 449.07±104.78 | P>0.05 |
| p__unclassified_d__Eukaryota | 43.91±10.2 | 85.41±43.81 | 18.6±3.62 | P>0.05 |
| p__Oomycota | 24.66±11.09 | 22.3±13.75 | 13.72±10.61 | P>0.05 |
| p__Olpidiomycota | 30.03±4.95 | 23.57±9.49 | 31.42±6.44 | P>0.05 |
| p__Chytridiomycota | 6.48±4.17 | 51.89±39.26 | 11.59±4.45 | P>0.05 |
| p__Zoopagomycota | 10.31±1.96 | 15.13±5.52 | 15.05±3.89 | P>0.05 |
| p__Thaumarchaeota | 15504.62±4364.31 | 7996.46±1409.38 | 12707.25±1521.34 | P<0.05 |
| p__Euryarchaeota | 772.69±93.76 | 5486.55±697.28 | 1665±408.75 | P<0.05 |
| p__unclassified_d__Archaea | 408.17±75.26 | 423.71±138.91 | 171.21±37.88 | P<0.05 |
| p__Candidatus_Thermoplasmatota | 249.65±30.87 | 713.94±367.98 | 317.39±45.87 | P>0.05 |
| p__Crenarchaeota | 83.25±28.95 | 190.31±72.77 | 200.36±36.66 | P>0.05 |
| p__Candidatus_Bathyarchaeota | 141.58±7.59 | 251.83±25.96 | 176.53±18.68 | P<0.01 |
| p__Candidatus_Woesearchaeota | 58.62±10.23 | 61.57±15.17 | 31.46±7.85 | P<0.05 |
| p__Candidatus_Aenigmarchaeota | 38.04±9.74 | 41.45±16.26 | 28.19±8.47 | P>0.05 |
| p__Nanoarchaeota | 25.98±3.3 | 34.86±30.26 | 6.02±4.89 | P>0.05 |
| p__Candidatus_Thorarchaeota | 18.14±5.25 | 44.95±27.77 | 10±3.57 | P>0.05 |
| p__Candidatus_Lokiarchaeota | 10.13±6.61 | 19.75±9.33 | 10.9±2.96 | P>0.05 |
| p__Candidatus_Marsarchaeota | 13.6±1.01 | 12.88±0.95 | 13.22±5.15 | P>0.05 |
| p__Candidatus_Micrarchaeota | 9.18±1.65 | 30.8±3.07 | 8.27±7.36 | P<0.01 |
| p__Candidatus_Heimdallarchaeota | 5.91±3.25 | 20.56±10.24 | 3.74±2.52 | P<0.05 |
| p__Candidatus_Altiarchaeota | 9.19±3.9 | 19.18±12.29 | 2.3±2.47 | P>0.05 |

Supplementary Table 7 Microbial community composition in root at the phylum level

| Phylum | R_1 | R_5 | R_11 | P |
| --- | --- | --- | --- | --- |
| p__Proteobacteria | 697730.49±42453.07 | 732046.69±44925.57 | 887941.74±137922.22 | P>0.05 |
| p__Actinobacteria | 362629.62±5281.15 | 578580.89±36771.98 | 472171.98±123857.42 | P<0.05 |
| p__Acidobacteria | 312550.75±14754.19 | 129090.4±19171.52 | 163389.42±49802.15 | P<0.01 |
| p__Verrucomicrobia | 42138.71±16477.88 | 127652.28±24845.6 | 133909.01±66878.68 | P>0.05 |
| p__Candidatus_Rokubacteria | 3498.89±898.34 | 60231.33±10972.38 | 27581.73±17758 | P<0.01 |
| p__Gemmatimonadetes | 54351.25±7310.17 | 21874.21±5706.01 | 24839.35±5727.67 | P<0.01 |
| p__Chloroflexi | 37230.44±5984.25 | 57104.9±13039.41 | 38966.27±8859.02 | P>0.05 |
| p__Bacteroidota | 67920.31±11039.05 | 31858.87±3044.94 | 37617.95±2967.45 | P<0.05 |
| p__Planctomycetota | 17887.71±2736.38 | 21979.81±2189.28 | 11073.95±2310.01 | P<0.01 |
| p__unclassified_d__Bacteria | 8571.59±949.12 | 14214.79±753.47 | 10656.25±1339.67 | P<0.01 |
| p__Firmicutes | 28737.01±12724.17 | 21495.39±10037.74 | 28150.04±22998.71 | P>0.05 |
| p__Nitrospirae | 5343.32±591.21 | 7572.25±2301.46 | 3873.82±2348.19 | P>0.05 |
| p__Cyanobacteria | 3479.32±282.83 | 8626.34±1236.26 | 4254±927.47 | P<0.01 |
| p__Candidatus_Eisenbacteria | 464.45±103.08 | 711.31±175.05 | 3371.05±1987.39 | P<0.05 |
| p__Candidatus_Tectomicrobia | 224.43±70.5 | 8674.15±3149 | 2354.69±1004.05 | P<0.01 |
| p__Ascomycota | 1803.14±367.36 | 862.62±12.62 | 5435.94±819.75 | P<0.05 |
| p__Basidiomycota | 5481.38±773.18 | 184.94±13.3 | 2387.34±461.41 | P<0.05 |
| p__Mucoromycota | 571.36±69.05 | 640.7±252.24 | 2949.23±149.42 | P<0.05 |
| p__unclassified_d__Eukaryota | 33.08±5.36 | 51.96±50.17 | 24.52±19.58 | P>0.05 |
| p__Oomycota | 67.61±1.81 | 81.96±73.15 | 62.38±14.41 | P>0.05 |
| p__Olpidiomycota | 17.43±8.15 | 12.99±4.71 | 29.01±11.65 | P>0.05 |
| p__Chytridiomycota | 6.17±3.15 | 41.37±59.38 | 12.24±6.08 | P>0.05 |
| p__Zoopagomycota | 13.18±2.55 | 20.95±2.78 | 17.72±7.54 | P>0.05 |
| p__Thaumarchaeota | 13115.57±5447.85 | 51900.02±7884.6 | 11750.44±7517.09 | P<0.01 |
| p__Euryarchaeota | 595.07±59.32 | 2186.76±769.16 | 833.76±387.05 | P<0.05 |
| p__unclassified_d__Archaea | 255.69±46.82 | 564.02±69.13 | 131.19±44.13 | P<0.001 |
| p__Candidatus_Thermoplasmatota | 172.56±42.41 | 211.76±16.8 | 183.68±61.61 | P>0.05 |
| p__Crenarchaeota | 95.18±23.1 | 486.48±114.83 | 157.15±69.91 | P<0.01 |
| p__Candidatus_Bathyarchaeota | 104.9±15.82 | 282.44±25.89 | 141.16±56.2 | P<0.01 |
| p__Candidatus_Woesearchaeota | 53.53±15.14 | 54.29±11.27 | 28.43±9.94 | P>0.05 |
| p__Candidatus_Aenigmarchaeota | 20.47±10.81 | 45.69±6.31 | 15.26±6.07 | P<0.01 |
| p__Nanoarchaeota | 15.25±2.93 | 7.14±10.15 | 3.21±1.98 | P>0.05 |
| p__Candidatus_Thorarchaeota | 10.63±1.43 | 14.63±5.31 | 5.24±8.03 | P>0.05 |
| p__Candidatus_Lokiarchaeota | 10.67±1.5 | 17.84±11.29 | 10.12±6.95 | P>0.05 |
| p__Candidatus_Marsarchaeota | 8.85±1.85 | 7.84±7.41 | 5.81±3.85 | P>0.05 |
| p__Candidatus_Micrarchaeota | 4.95±3.03 | 9.4±3.86 | 6.85±1.82 | P>0.05 |
| p__Candidatus_Heimdallarchaeota | 8.75±2.91 | 10.15±5.97 | 5.75±1.38 | P>0.05 |
| p__Candidatus_Altiarchaeota | 3.92±2.68 | 3.91±1.43 | 2.93±2.69 | P>0.05 |

Supplementary Table 8 Nutrient contents of non-rhizosphere soil and rhizosphere soil

|  | RS_1 | RS_5 | RS_11 | PS_1 | PS_5 | PS_11 |
| --- | --- | --- | --- | --- | --- | --- |
| TP  (g/kg) | 0.30±0.02a | 0.24±0.00b | 0.27±0.01ab | 0.32±0.01a | 0.32±0.01a | 0.21±0.01b |
| SOC  (g/kg) | 11.16±0.15c | 19.15±0.32a | 15.21±0.55b | 15.64±0.05c | 22.85±0.32a | 17.22±0.11b |
| NH_4_^+^  (mg/kg) | 0.52±0.02c | 1.12±0.06a | 0.95±0.05b | 0.29±0.01b | 0.50±0.01a | 0.53±0.02a |
| NO_3_^-^  (mg/kg) | 14.19±0.09a | 7.69±0.11b | 2.77±0.06c | 8.79±0.04a | 6.78±0.17b | 2.62±0.15c |
| TN  (%) | 0.10±0.00b | 0.15±0.00a | 0.09±0.00b | 0.10±0.01b | 0.17±0.00a | 0.09±0.01b |
| TC  (%) | 2.16±0.04b | 2.80±0.01a | 2.15±0.04b | 2.09±0.02b | 3.11±0.01a | 2.11±0.02b |
